# The association between statin use and osteoarthritis-related outcomes: An updated systematic review and meta-analysis

**DOI:** 10.3389/fphar.2022.1003370

**Published:** 2022-11-24

**Authors:** Zhan Zhang, Chunbo Deng, Xun Ma, Qijun Wu, Fenghua Zhou, Xueyong Liu

**Affiliations:** ^1^ Department of Orthopedics, Shengjing Hospital of China Medical University, Shenyang, China; ^2^ Department of Orthopedics, Central Hospital of Shenyang Medical College, Shenyang, China; ^3^ Department of Rehabilitation, Shengjing Hospital of China Medical University, Shenyang, China; ^4^ Department of Clinical Epidemiology, Shengjing Hospital of China Medical University, Shenyang, China

**Keywords:** statin, osteoarthritis, meta-analysis, risk, antihypertensive drugs

## Abstract

**Objective:** Findings among studies evaluating the effect of statin use and OA development in a 2020 meta-analysis of data from 11 observational studies of statin use and osteoarthritis (OA) revealed controversial results. We aimed to determine the associations between statin use and OA-related outcomes in an updated meta-analysis.

**Methods:** The protocol was registered with PROSPERO (CRD42020163983). A systematic literature retrieval was performed in the online databases, including PubMed, Cochrane Library, Embase, Web of Science, and Scopus, from inception to 1 June 2022, for clinical studies that compared the effects of statin users vs. nonusers on OA-related outcomes risks. Systematic reviews and meta-analyses were performed to estimate the correlations between statin use and OA-related outcomes. Tendency analysis was also used to estimate dose-response effects. The risk of bias was evaluated with the Newcastle–Ottawa scale.

**Results:** We included 23 studies involving more than 6,000,000 participants. Statin use was associated with increased OA risk (OR 1.099 [95%CI 1.002–1.206, *p* = 0.045]). Higher statin doses had higher OA risk (simvastatin equivalent daily of >40 mg). OA and related surgery risks were significantly reduced in statin users using antihypertensive drugs (AHDs). No significant differences were seen in other outcomes.

**Conclusion:** This meta-analysis inferred that statin use might be associated with increased OA development, especially at higher doses. The present study highlights the importance of recognizing potential OA risk in the population with long-term and/or high-dose statin use, especially in older populations. In addition, AHDs are associated with lower OA risk and fewer surgeries in hypertensive statin users. Due to limitations of heterogeneity and confounders, more rigorous studies are needed to define the correlations between statin use and OA-related outcomes.

## Introduction

Osteoarthritis (OA) is the most common degenerative joint disease, and the major cause of joint pain and disability. OA is increasingly recognized as worldwide health concern due to low quality of life and huge social and economic burden (Hunter and Bierma-Zeinstra, 2019; Katz Jeffrey et al., 2021). OA pathogenesis is complex and remains largely unclear. In the late stage of OA, surgeries are effective but have a limited long-term prognosis and prosthesis life (Evans et al., 2019). Therefore, early pharmacological intervention should be considered, but challenges remain due to limited analgesic outcomes and the occurrence of adverse events (Gregori et al., 2018). Metabolic factors like dyslipidemia were confirmed as being significantly associated with OA (Mobasheri et al., 2017; Choi et al., 2019; Song et al., 2021). The proinflammatory effects of lipids, adipokine-linked proinflammatory cytokines and pathways have been reported to be associated with OA pathogeneses (Gkretsi et al., 2011; Neumann et al., 2016). Therefore, lipid-related metabolisms and pathways could be attractive targets for OA management.

Statins are currently the most effective drugs used as lipid-lowering agents. In clinical practice, statins are commonly prescribed in cardiovascular diseases and dyslipidemia treatments, especially in older populations with multiple geriatric and metabolic diseases. In addition, statins have been shown to function as agents of anti-inflammation, offering cartilage protection (Ridker et al., 2005; Gkretsi et al., 2011). Hence, there is a growing interest in statins as potential disease-modifying agents for OA (Baker et al., 2011; Gkretsi et al., 2011). However, statin use is also reported to be linked to adverse musculoskeletal and metabolic effects, but this conclusion remains controversial (Mansi et al., 2013; Thompson et al., 2016).

A 2020 meta-analysis looking at the association between statin use and OA development and progression provided controversial results due to multiple limitations (Wang et al., 2020). Meanwhile, multiple OA-related outcomes, such as OA surgical risks, remain to be investigated. It is unclear if statin use is associated with OA-related outcomes, although some studies have reported inspiring outcomes (Clockaerts et al., 2012; Valdes et al., 2014; Michaëlsson et al., 2017; Haj-Mirzaian et al., 2019; Veronese et al., 2019). Therefore, the purpose of this study was to conduct a systematic review and meta-analysis to estimate the effects of statin use on OA-related outcomes.

## Methods

### Search strategy and selection criteria

This study was performed according to the Preferred Reporting Items for Systematic Reviews and Meta-Analysis (PRISMA) guidelines (Liberati et al., 2009) and guidance from the Cochrane Collaboration (Higgins et al., 2019), and the protocol was registered in with PROSPERO (CRD42020163983). The PRISMA checklists and amendments from the primary version were available in Supplementary Data. A systematic online search was conducted using electronic databases, including PubMed, Cochrane Library, Embase, Web of Science, and Scopus, without language limitations, from inception to 1 June 2022. The online retrieval was performed with the terms: “osteoarthritis OR degenerative joint disease” and “statin.” The detailed search strategy is provided in Supplementary Data. The first search was performed on 1 January 2020 and updated on 1 June 2022.

Study selection was based on the PICOS statement. *Population/patient:* participants using statins with controls from the same population without statin medication prior to identification; *intervention:* statin use; *comparison:* statin users vs. nonusers on OA-related outcomes; *outcome:* data concerning OA-related outcomes, including statin-related OA risk; OA-related surgery; duration and dosage of statin use and OA risk; *study design:* clinical randomized/case-control/cohort studies. Non-clinical studies, studies with potential bias in participant populations, or studies without available data or conclusion (including reviews, protocols, etc.) were excluded. This part of the study was independently performed by two investigators in duplicate and double-checked by a third investigator. Discordant judgments were addressed by open discussion with the research team and resolved by consensus.

### Data extraction and methodologic quality assessments

Two investigators independently performed the data extraction and quality assessments. From each included study, we extracted information such as demographic data, conclusions and clinical significance. If multiple studies were analyzed with potential overlapping participants, we retained studies with the most detailed methods, the most participants and the most adjusted confounding factors. If multiple non-overlapping data were found, the data were allowed and regarded as independent.

The risk of bias of included studies was assessed with the Newcastle–Ottawa scale (NOS) (Wells et al., 2000), which evaluates studies with >7 stars or those with greater than median stars to be of high quality with low risk for bias.

### Data analysis

Data of statin users vs. nonusers on OA-related outcomes risks were calculated, including data concerning OA-related outcomes, including statin-related OA risk; OA-related surgery; duration and dosage of statin use and OA risk. OA diagnoses were defined by radiologic (Kellgren-Lawrence grade ≥2) or clinical evidence, and endpoint events were defined as OA-related outcome events. Propensity score-matched data were preferred for the entire analysis, then data of the largest number of confounders adjusted, followed by raw data. Ratios and eligible data were used. Hazard ratios (HRs), odds ratios (ORs) and relative ratios (RRs) in this study were used, deemed as homogenous and analyzed together.

When combination or transformation of study estimation values were needed, in the situations like multiple co-existing estimations obtained from the same reference control group, the method developed by Hamling’s study was used when participant numbers at every level were available (Hamling et al., 2008). Otherwise the method presented in Gao’s study was used (Gao et al., 2018). If transformations were not applicable, estimations using the most participants, the longest durations, or the highest doses were used. When two estimates were compared, and relative OR value and 95% CI were to be used, the method presented in Altman and Bland (2003) was used.

Meta-analyses were performed using the meta package for R software (version 3.6.3) (Balduzzi et al., 2019). Heterogeneity was identified by applying the DerSimonian–Laird method of the Q-test and was quantified using I^2^ values. Pooled data had low heterogeneity if *p* was greater than 0.1 and I^2^ was less than 50%. In this case, a fixed-effects model was used; otherwise, a random-effects model was used (Egger et al., 1997; Higgins et al., 2003). Statistical analyses were two-sided, and a *p*-value less than 0.05 was considered significant.

Sensitivity analysis were performed to reduce heterogeneity and detect additional potential correlations. Subgroup analyses were performed in the overall cohorts where significant heterogeneity was detected and were based on extracted data. Egger’s linear regression and Begg’s rank correlation tests were performed to evaluate potential publication bias for outcomes with more than 10 included studies (Begg and Mazumdar, 1994; Sterne et al., 2000; Mathur Maya and VanderWeele Tyler, 2020).

For data deemed ineligible for meta-analyses, we listed and re-analyzed all data without statistical syntheses; however, the strengths of this evidence were considered to be inferior to those of the meta-analyses.

## Results

### Literature identification and data characteristics

**FIGURE 1:**
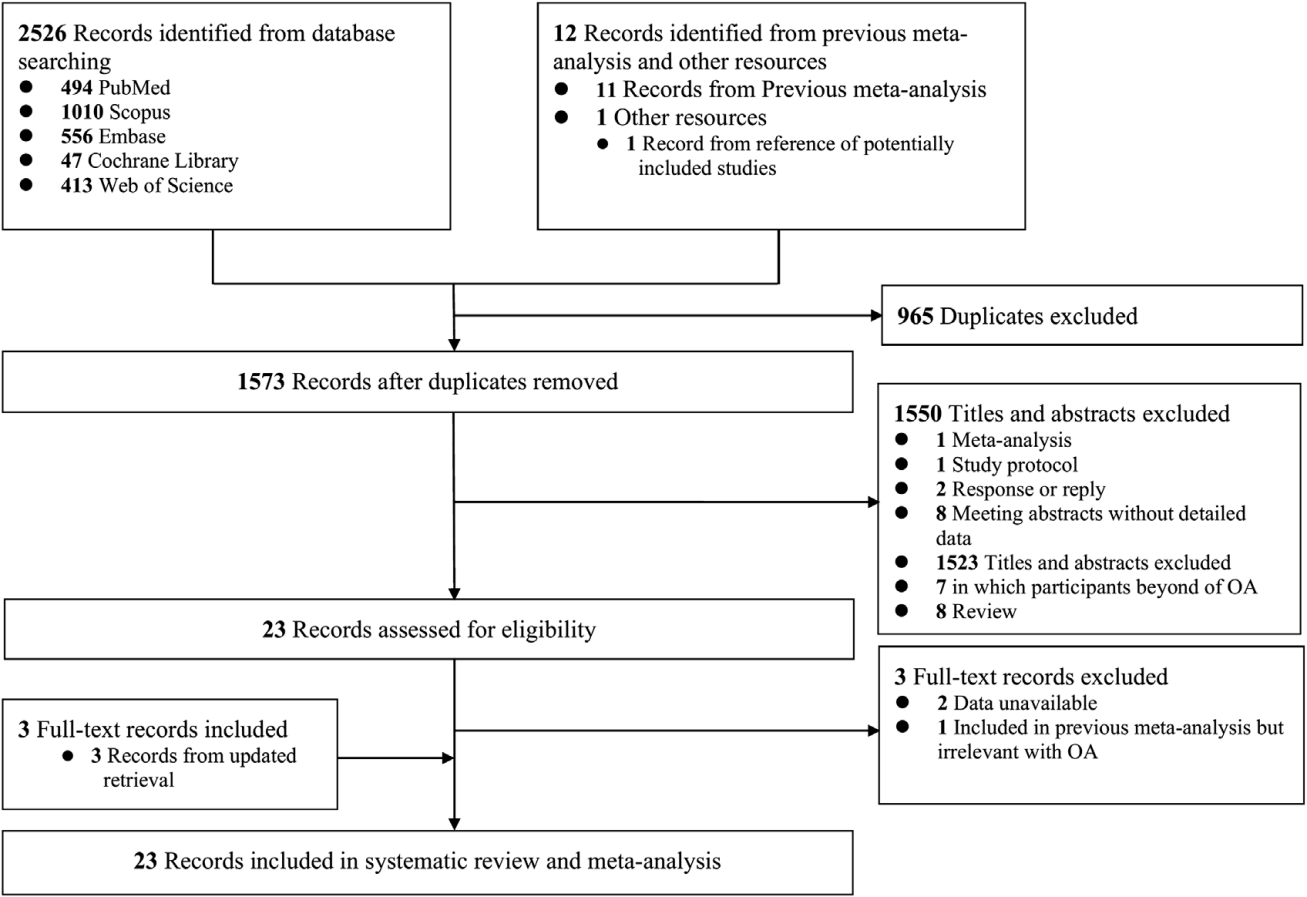
Flow diagram of literature search.

The systematic literature search originally retrieved 1574 unique citations. A total of 23 studies (20 cohort studies and three case-control studies) met the eligibility criteria and were included in this study (Figure 1) (Beattie et al., 2005; Chodick et al., 2010; Chaganti et al., 2012; Clockaerts et al., 2012; Kadam et al., 2013; Mansi et al., 2013; Riddle et al., 2013; Cemeroglu et al., 2014; Valdes et al., 2014; Frey et al., 2017; Garcia-Gil et al., 2017; Michaëlsson et al., 2017; Roy et al., 2017; Burkard et al., 2018; Cheng et al., 2018; Eymard et al., 2018; Haj-Mirzaian et al., 2019; Jonsson et al., 2019; Veronese et al., 2019; Cook et al., 2020; Sarmanova et al., 2020; Milena et al., 2021; Perry et al., 2021). Most of these studies were from famous research cohorts or databases, like the Clinical Practice Research Datalink (CPRD) and the Osteoarthritis Initiative (OAI) comprising more than 6,000,000 participants. Detailed characteristics of the included studies and participants are shown in Table 1. Due to the very large amount of basic data, we listed the data sources and potentially duplicate data in Supplementary Table S1. Assessments of methodologic quality according to NOS are summarized in Supplementary Table S2, and most studies scored above 6 of 9.

**TABLE 1:** Characteristics of included studies.

Included study	Data resource	Study design	Lesion	Definition of statin use	Date range	Statin users/Nonusers	Mean age (year, mean ± SD)		Gender (female/male)		Follow-up (year)	Confounder adjusted
							Statin users	Nonusers	Statin users	Nonusers		
Frey et al. (2017)	CPRD	Population-based case-control study	Hand	Prescription for a statin within 365 days prior to the index date	1995.01–2014.12	19590/19590	62.4		15019/4571	15019/4571	NA	Charlson comorbidity index, Co-medication, Cigarette and/or alcohol consumption
Eymard et al. (2018)	SEKOIA trial	Post-hoc analysis of RCT	Knee	Intake of statin at baseline	2006.04.15–2011.03.30	71/265	64.0 ± 6.9	62.3 ± 7.3	46/25	188/77	3	Charlson comorbidity index
Valdes et al. (2014)	GOAL	Case-control study	Hand, hip and knee	NA	2002–2006	661/2510	68.8 ± 6.52	65.96 ± 8.10	249/412	1288/1222	NA	Demographic data, Charlson comorbidity index, Co-medication, Cigarette and/or alcohol consumption
Beattie et al. (2005)	Study of Osteoporotic Fractures	Multicenter cohort study	Hip	Taking any available prescription oral statin	1986.09–1988.10	386/5292	70.6 ± 4.6		5678/0		8	Demographic data, Charlson comorbidity index, Co-medication
Jonsson et al. (2019)	AGES-Reykjavik study	Population-based, multidisciplinary longitudinal cohort study	Hand	NA	2002–2006	1101/3656	76 ± 5		2714/2043		5	Demographic data, Charlson comorbidity index, Co-medication, and Others
Chodick et al. (2010)	Maccabi Healthcare Services	Retrospective cohort study	NA	At least one dispensed prescription of statin	1998–2007	138992/54378 (PDC<20%)	56.43 ± 12.87		95132/98638		4.42–5.06	Demographic data, Charlson comorbidity index, Co-medication, and Others
Riddle et al. (2013)	OAI	Longitudinal cohort study	Knee	A cumulative use of more than 120 days and/or a daily intake of more than 50% of the recommended daily adult dose	2004.02–2006.05	448/1759	62.5 ± 9.0	60.7 ± 9.3	248/200	1062/697	4	Demographic data, Charlson comorbidity index, and Others
Kadam et al. (2013)	GPRD	Cohort study	NA	A minimum duration of 2 years of statin use	1995.01.01–1996.12.31	4976/11633	65 ± 9.6	70 ± 13.1	8282/8327		10	Demographic data, Charlson comorbidity index, Co-medication
Cheng et al. (2018)	NHIRD	Retrospective cohort study	Spinal joint	Received any statin treatment before 1 January 2001	2001–2010	7238/164454	40–65		82961/88731		7	Demographic data, Charlson comorbidity index, and Others
Garcia-Gil et al. (2017)	Chingford Women’s Study	Prospective cohort study	Hand	NA	1988–1989	8/269	50.4 ± 4.8		277/0		11	Demographic data, Co-medication
Veronese et al. (2019)	OAI	Longitudinal cohort study	Knee	Prescription of a certain statin	2004.02–2006.05	1127/3321	64.3 ± 8.4	60.0 ± 8.2	53.1%/46.9%	59.8%/40.2%	4	Demographic data, Charlson comorbidity index, Co-medication, and Others
Burkard et al. (2018)	CPRD	Retrospective cohort study	Hand	With ≥1 new prescription for a certain statin after a statin-free period of ≥3 years	1996.01–2015.12	237864/6020144	62.7 ± 9.4	58.0 ± 10.1	116938/120926	3483217/2536927	Up to 5.5	Demographic data, Charlson comorbidity index, Co-medication, Cigarette and/or alcohol consumption
Roy et al. (2017)	Electronic medical record	Retrospective cohort study	Knee	NA	2015.07.01–2015.12.31	720/2780	46.9 ± 17.6		1910/1590		NA	NA
Clockaerts et al. (2012)	Rotterdam study	Prospective cohort study	Knee	A cumulative use of more than 120 days and/or a daily intake of more than 50% of the recommended daily adult dose	1990–1993	317/2604	64.3 ± 8.4	60.0 ± 8.2	178/139	1495/1109	Average 6.5	Demographic data, Charlson comorbidity index, Co-medication, and Others
Michaëlsson et al. (2017)	MDCS	Cohort study	Hip and/or knee	Any use of statin in a specified time	1991–1996	9460/15525	69.5 ± 7.0	67.9 ± 7.8	15491/9494		2005.07.01–2011.12.31 or outcome	Demographic data, Charlson comorbidity index, Cigarette and/or alcohol consumption, and Others
	MPP				2002–2006	7111/9586	69.6 ± 6.1	68.5 ± 6.7	6052/10645			
	SMC				1987–1990	14788/36140	69.1 ± 7.9	69.5 ± 9.6	50928/0		2005.07.01–2012.12.31 or outcome	
	COSM				1997	14153/25558	69.5 ± 8.5	66.6 ± 9.7	0/39997			
Haj-Mirzaian et al. (2019)	OAI	Longitudinal cohort study	Knee	Regular statin use before enrollment	2004.02–2006.05	1698/4408	66.0 ± 7.6	62.3 ± 9.0	980/718	2812/1696	8	NA
Cemeroglu et al. (2014)	Hospital record	Case-control study	Hand	NA	NA	17/44	65.46 ± 8.0		61/0		NA	NA
Cook et al. (2020)	CPRD	Retrospective Cohort Study	Hip and/or knee	Continuous statin exposure at a given time	1988.01.01–2016.12.31	65032/86273	70.3 ± 8.5	69.2 ± 10.8	34942/30090	54297/31976	3.9	Demographic data, Charlson comorbidity index, Co-medication
Chaganti et al. (2012)	MOST	Population-based cohort study	Knee	Continuous use at both the baseline and 30-month visit	NA	432/1243	50–79		960/715		2.5	Demographic data, Charlson comorbidity index
Sarmanova et al. (2020)	CPRD	Retrospective cohort study	Hip and/or knee	People ever prescribed a statin (two or more prescriptions)	1987.01.01–2017.07.31	562526/562526	63.03 ± 11.02	63.42 ± 11.11	266324/196202	266324/196202	6.25–6.88	Demographic data, Charlson comorbidity index, Co-medication, Cigarette and/or alcohol consumption
Perry et al. (2021)	OAI	Longitudinal cohort study	Knee	Medication exposure in the previous 30 days	2004.02–2006.05	548/1455	63.3 ± 8.98		1115/888		Up to 8	Demographic data, Charlson comorbidity index
Milena et al. (2021)	LEGS study	Longitudinal study	Knee	Any regular medication taken during the previous 7 days	NA	131/367	60 ± 8		282/216		1–2	Demographic data, Charlson comorbidity index, Co-medication
Mansi et al. (2013)	San Antonio Military Multimarket area	Retrospective cohort study	NA	At least 1 dispensed statin prescription of a 3-month supply	2003.10.01–2010.03.05	12980/45997	67 ± 13/44 ± 15		41%/59% + 56%/44%		4	Demographic data, Charlson comorbidity index, Co-medication, Cigarette and/or alcohol consumption

Abbreviation: CPRD, UK-based Clinical Practice Research Datalink; SU, statin users; NU, nonusers; GOAL, Genetics of OA and Lifestyle; SEKOIA, Strontium ranelate Efficacy in Knee OsteoarthrItis triAl; AGES-Reykjavik, Age, gene/environment susceptibility-Reykjavik; OAI, Osteoarthritis Initiative; GPRD, General Practice Research Database; NHIRD, National Health Insurance Research Database; MDCS, The Malmö Diet and Cancer Study; MPP, The Malmö Preventive Project; SMC, Swedish Mammography Cohort; COSM, Cohort of Swedish Men; DNHS, Danish National Health System; NA, not available; LEGS study, Long-term Evaluation of Glucosamine Sulfate study; PDC, proportion of days covered.

### Statistical analyses

Based on the extracted data and transformed data, statistical analyses were performed in several cohorts, including OA incident risk (risk cohort), OA-related surgery risk (surgery cohort), duration and dosage of statin use and OA incident risk (duration/dosage cohort), OA progression risk (progression risk) and antihypertension drug and statin co-use and OA incident/surgery risk (AHD cohort). The cohort outcomes and evidence analysis of non-meta-analyzed studies are displayed in Tables 2, 3.

**TABLE 2:** Results of Meta-analysis of the correlations between statin use and OA-related outcomes.

Group	Number of studies	OR (95%CI)	*p*-value	I^2^ (%)	Publication bias (*p*)	
					Begg	Egger
Risk analysis						
Adjusted estimations	11	1.099 (1.002–1.206)	0.045	79.60	0.734	0.377
• **Adjusted OR values**	**4**	**1.146 (1.090–1.204)**	**<0.001**	**0.00**		
• **Adjusted RR values**	**2**	**1.331 (1.234–1.436)**	**<0.001**	**0.00**		
• Adjusted HR values	9	1.086 (0.973–1.206)	0.121	83.00		
Adjusted estimations^a^	3	0.965 (0.904–1.029)	0.278	0.00		
Raw estimations	11	1.247 (0.988–1.573)	0.063	97.40	0.815	0.416
Progression analysis						
Follow-up vs. baseline	5	1.034 (0.798–1.341)	0.800	0.00		
Adjusted estimations	6	0.867 (0.557–1.349)	0.527	78.80		
Duration analysis						
OA incidence estimations						
**Former statin use**	**4**	**1.169 (1.022–1.338)**	**0.023**	**0.00**		
0–1 year vs. nonusers	7	1.077 (0.992–1.170)	0.077	34.40		
0–2 years vs. nonusers	7	1.066 (0.998–1.138)	0.057	0.00		
0–3 years vs. nonusers	7	1.030 (0.979–1.084)	0.257	0.00		
0–4 years vs. nonusers	7	1.030 (0.979–1.084)	0.257	0.00		
0–5 years vs. nonusers	7	1.032 (0.979–1.085)	0.241	4.90		
0–6 years vs. nonusers	7	1.035 (0.987–1.086)	0.152	3.50		
>1-year vs. nonusers	8	1.030 (0.982–1.081)	0.219	34.20		
>2-year vs. nonusers	8	1.019 (0.965–1.076)	0.493	28.10		
>3-year vs. nonusers	7	1.064 (0.985–1.150)	0.104	14.20		
>4-year vs. nonusers	3	1.073 (0.949–1.214)	0.260	0.00		
Duration tendency estimation of OA incidence						
Tendency from 0–1 to 0–6 years	6	1.006 (0.974–1.039)	0.706	0.00		
Tendency from 0–1 to >4 years	5	1.008 (0.872–1.166)	0.490	7.60		
**Overall tendency in** Michaëlsson et al. (2017)^b^	**16**	**1.038 (0.971–1.110)**	**0.272**	**30.70**		
>4 years vs. 0–4 years		1.042 (0.912–1.190)				
>3 years vs. 0–3 years		1.033 (0.942–1.133)				
>2 years vs. 0–2 years		0.956 (0.878–1.041)				
>1 year vs. 0–1 year		0.956 (0.869–1.052)				
Dose analysis						
**Overall Outcomes**						
Low	6	1.008 (0.945–1.076)	0.807	0.00		
Low-medium	4	1.044 (0.990–1.102)	0.115	0.00		
Medium	4	1.063 (0.995–1.136)	0.070	0.00		
**Medium-high**	**4**	**1.079 (1.012–1.151)**	**0.021**	**0.00**		
**High**	**4**	**1.383 (1.054–1.815)**	**0.019**	**0.00**		
**Total tendency 1 (Every higher vs. adjacent lower one)**	**18**	**1.039 (1.000–1.080)**	**0.049**	**0.00**		
Total tendency 2 (Including lowest dose vs. nonusers)^c^	22	1.035 (0.999–1.072)	0.060	0.00		
Total tendency 3 (Including Kadam et al. (2013)^d^	8	1.034 (0.999–1.071)	0.057	0.00		
**Overall >20 mg/d vs. <20 mg/d of statin use** ^d^	**9**	**1.108 (1.004–1.222)**	**0.041**	**0.00**		
**Outcomes of statin use with antihypertensive drugs prescribed**						
Low	4	0.966 (0.914–1.021)	0.204	0.00		
Low-medium	4	0.932 (0.836–1.041)	0.215	0.00		
Medium	4	0.972 (0.902–1.046)	0.446	0.00		
Medium-high	4	0.988 (0.922–1.058)	0.720	0.00		
High	4	1.252 (0.934–1.677)	0.133	16.20		
Total tendency^c^	20	1.005 (0.953–1.059)	0.858	0.00		
AHDs use in OA risk						
**EST-1**	**4**	**0.901 (0.825–0.984)**	**0.020**	**0.00**		
**EST-2**	**12**	**0.906 (0.837–0.981)**	**0.015**	**0.00**		
OA-related surgeries						
Overall estimation	5	0.982 (0.950–1.014)	0.263	0.00		
Michaëlsson et al. (2017)	4	1.036 (0.960–1.117)	0.368	0.00		
**EST-1 (AHD)**	**4**	**0.912 (0.833–0.999)**	**0.048**	**0.00**		
**EST-2 (AHD)**	**4**	**0.882 (0.783–0.994)**	**0.039**	**0.00**		
**Raw values**	**5**	**1.216 (1.190–1.242)**	**<0.001**	**42.90**		

^a^ People in this group of participants did not have dyslipidemia or hyperlipidemia.

^b^ In this study, all the estimations of every duration vs. adjacent shorter duration were meta-analyzed.

^c^ This cohort was calculated by meta-analyzing the ratios of estimations of a higher dose vs. an adjacent lower dose.

^d^ This cohort was calculated by meta-analyzing the meta-analyzed ratios of estimations of a higher dose vs. an adjacent lower dose in every study.

Abbreviation: OR, odds ratio; HR: hazard ratio; OA, osteoarthritis; AHDs, antihypertensive drugs; EST-1 and -2, estimation-1 and -2: these two cohorts of antihypertensive drugs analysis are described in the text.

Bold values indicate statistical significance (*p*<0.05).

### OA risk and OA-related surgery risks associated with statin use

**FIGURE 2:**
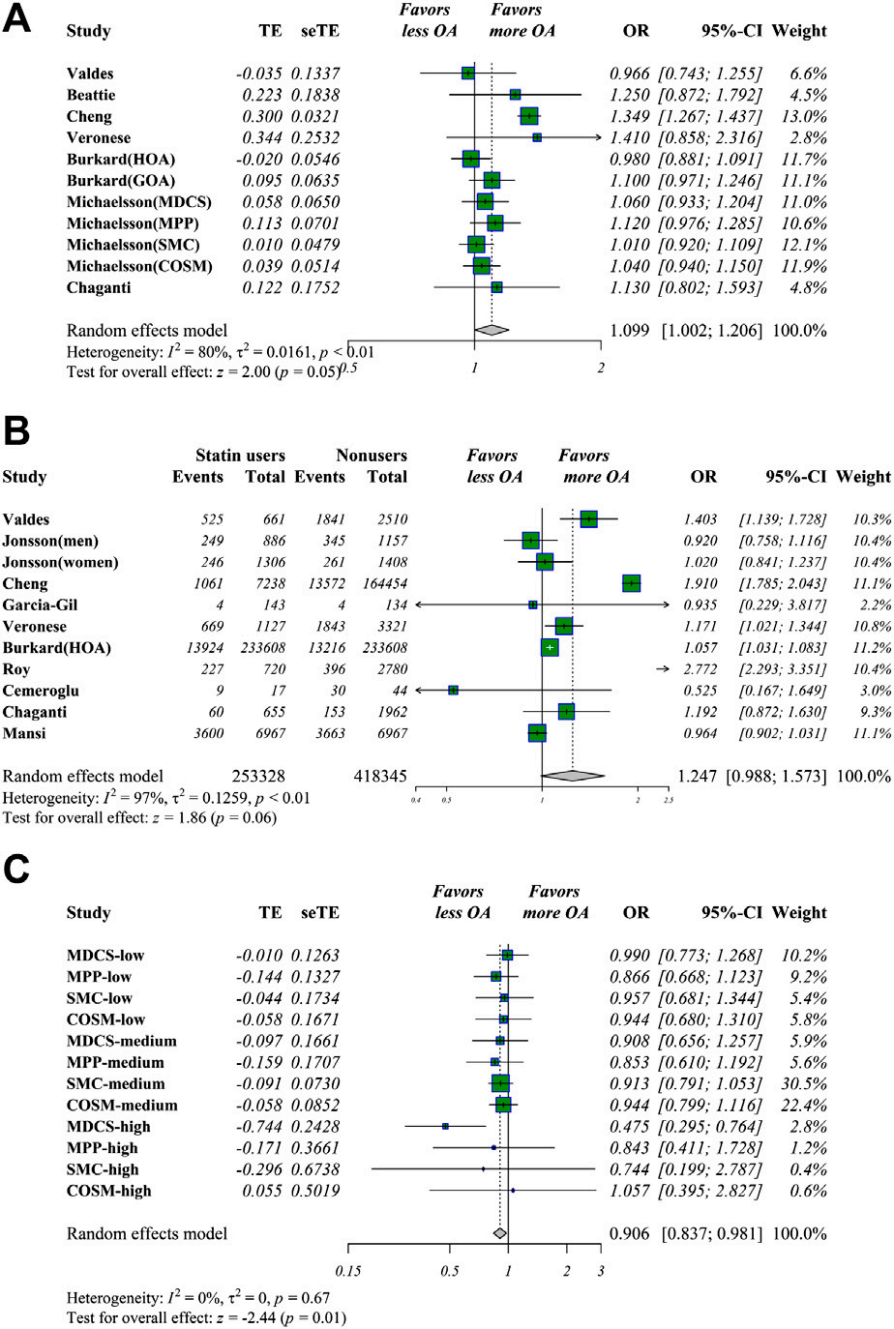
Meta-analysis of estimations of statin use and OA risk. Meta-analysis of overall adjusted estimations **(A)** and raw data **(B)** of statin users vs. nonusers for OA risk. Statin use was linked to significantly higher OA risk in adjusted estimations in adjusted OR values and raw data **(C)** Meta-analysis of potential impacts of antihypertensive drugs (AHDs) on hypertensive statin users. AHD use may be linked to decreased OA risk in hypertensive statin users.

Of the 23 included studies, 12 had estimated statin use and OA risk in hazard risk, odds ratio, and relative risk (HR/OR/RR) data and raw numbers. Statin use was detected as being associated with slightly higher OA risk in overall adjusted estimations (OR 1.099 [95% CI 1.002–1.206, *p* = 0.045]). Similar outcomes were seen in the raw data (OR 1.247 [95% CI 0.988–1.573, *p* = 0.063]) (Figures 2A,B). After non-meta-analyzed evidence was re-analyzed, no consistent conclusion was obtained about statin use and OA risk, but statin use may increase knee pain and function loss (Table 3).

**TABLE 3:** Re-analysis of the data ineligible for meta-analyses.

Study	Reason for non-inclusion in the meta-analysis	Conclusion	Evidence estimation and/or re-analysis
Risk cohort			
Riddle et al. (2013)	Multiple studies were involved in potential duplicate data in the database osteoarthritis initiative (OAI). This study was deemed not preferred for use in the meta-analysis, as described in Supplementary Table S1.	Statin use was not associated with improvements in knee pain, function or structural progression.	The radiologic diagnosis defined in the study of Riddle was Kellgren–Lawrence grade 1 or greater, and no significant association was found between statin use and each-leg OA (*p* = 0.85 and 0.36). When the radiologic diagnosis definition was set as Kellgren–Lawrence grade 2 or greater, statin use may reduce OA risk in the right-leg cohort (OR 0.511 [95%CI 0.408–0.641], *p* < 0.0001).
			However, we re-analyzed the 7-day pain rating, Western Ontario and McMaster Universities Osteoarthritis Index (WOMAC) pain scale and physical function. Statin use was not associated with improvement of knee pain and function in the primary conclusion. The standardized mean differences (SMDs) of statin users vs. nonusers were calculated and meta-analyzed from baseline to the 4-year group. Statin use may be linked to increased knee pain in 7-day pain rating (SMD 0.147 [95%CI 0.099–0.195, *p* < 0.001]), WOMAC pain (SMD 0.178 [95%CI 0.131–0.226, *p* < 0.001]), and decreased WOMAC function (SMD 0.174 [95%CI 0.126–0.222, *p* < 0.0001]).
Chodick et al. (2010)	In this study, participants with a proportion of follow-up days covered (PDC) 0%–20% of statin use were defined as the control group without rigorously defining nonusers.	The relationship between continuation of statin use and OA onset was weak and limited to patients with short-term follow-up.	Based on provided numbers of participants of PDC levels (<20% (References), 20%–39%, 40%–59%, 60%–79% and >80%), every PDC level of statin use vs. reference level was calculated. A significant elevation of OA risk was detected in >20% PDC vs. <20%, OR 1.218 [95%CI 1.176–1.262, *p* < 0.001].
			We meta-analyzed the HRs of the rest levels vs. references following the method of Gao et al. (2018) in the >1-year follow-up group and the >5-year follow-up group. Statin use was observed to be associated with a decreased OA risk in the >1-year follow-up group (OR 0.883 [95%CI 0.851–0.916], I^2^ = 58.20%), but OA risk was increased in the >5-year follow-up group (OR 1.065 [95%CI 1.016–1.116, *p* < 0.001], I^2^ = 0.00%).
Mansi et al. (2013)	The propensity score–matched (PSMed) data at study baseline were used in the meta-analysis, but outcome data in this study were not meta-analyzed. The study is listed here because outcome data were described as “osteoarthritis/arthropathies” instead of OA.	Musculoskeletal conditions, arthropathies, injuries, and pain are more common among statin users than among similar nonusers.	The outcome estimations were PSMed cohort (no confounder adjusted), all-patients cohort, no-Charlson-comorbidities cohort, and sensitivity analysis of musculoskeletal incident cohort (OA participants at baseline excluded) and 2-year cohort (statin use < 2-year). Statin use was detected as being significantly associated with increased OA/arthropathies risk in the all-patients cohort (OR 1.07 [1.01–1.15, *p* = 0.03]), the no-Charlson-comorbidities cohort (OR 1.10 [1.02–1.19, *p* = 0.01]), the musculoskeletal incident cohort (OR 1.10 [1.01–1.20, *p* = 0.03]) and the 2-year cohort (OR 1.08 [1.005–1.15, *p* = 0.04]), and close to significant in the PSMed cohort (OR 1.07 [95%CI 0.99–1.16, *p* = 0.07]).
Clockaerts et al. (2012)	In this study, participants with less than 50% of the recommended dose of statin use and nonusers were defined as the control group, and rigorous nonusers were needed in the control group.	Statin use is associated with more than a 50% reduction in the overall progression of OA of the knee but not of the hip.	In this study, patients’ hips and knees (two each) were calculated individually and were thus deemed as an independent cohort to be meta-analyzed. Based on the provided raw data, statin use was detected as being associated with a decreased OA risk (OR 0.613 [95%CI 0.488–0.769, *p* < 0.001], I^2^ = 0.00%]) at baseline timepoint and (OR 0.659 [0.539–0.805, *p* < 0.001], I^2^ = 0.00%) at the follow-up timepoint.
Progression cohort			
Perry et al. (2021)	In this study, the main outcome was medial minimum joint space width (mJSW), so the data could not be used along with that from other studies in this cohort.	There was no statistically significant association between change in mJSW and current statin use.	In this study, statin use was not associated with increased mJSW loss (unstandardized beta coefficients 0.034, [95%CI -0.08–0.01, *p* = 0.11]). Statin use was also not associated with radiologic OA progression.
Duration cohort			
Frey et al. (2017)	Multiple studies were involved in potential duplicate data in the database osteoarthritis initiative (CPRD). This study was deemed not preferred for use in the meta-analysis, as described in Supplementary Table S1.	Hyperlipidemia may be an independent risk factor for new-onset hypertrophic OA (HOA).	Statin comedication within 1-year prior identification was associated with a higher risk of OA (OR 1.07 [95%CI, 1.01–1.13, *p* value unavailable]) versus nonusers. More or less than 1 year of statin use was associated with a significantly elevated OA risk with hyperlipidemia, but these estimations were obtained without comorbidity adjusted for hyperlipidemia.
Kadam et al. (2013)	Potential duplicate data with Burkard et al., 2018 (CPRD). This study was deemed not preferred for use in the meta-analysis, as described in Supplementary Table S1.	Higher statin dose and higher statin dose increments were associated with a reduction in clinically defined OA outcomes.	Larger increments in the dose of statins may decrease OA compared to nonusers over a 4-year time period. However, this outcome did not cover overall participants.
Riddle et al. (2013)	As above	Statin use was not associated with improvements in knee pain, function or structural progression over the 4-year study period.	SMDs and their 95% CI of the 7-day pain rating, Western Ontario and McMaster Universities Osteoarthritis Index (WOMAC) pain scale and physical function of statin users versus nonusers were calculated as above. Correlation coefficients and a fitting curve of SMDs and their 95%CI were calculated and visualized to test whether SMDs increased with statin use duration. Outcomes of 7-day pain rating (R = 0.56, *p* = 0.32), WOMAC pain scale (R = 0.32, *p* = 0.6) and physical function (R = 0.37, *p* = 0.54) were not associated with OA improvements.
Veronese et al. (2019)	This study was listed because the tendency of duration-estimation correlations was also analyzed.	A significantly lower risk of developing knee pain was observed in statin users for >5 years.	The tendency coefficients and a fitting curve were calculated and visualized from <1 month to >5 years. Duration of statin use was not associated with pain worsening (R = −0.33, *p* = 0.59), radiographic OA progression (R = 0.78, *p* = 0.12), or symptomatic OA progression (R = −0.73, *p* = 0.16).
Chodick et al. (2010)	The detailed numbers of every PDC level in the >1-year and the >5-years groups were unavailable.	The relationship between the continuation of statin use and OA onset was weak.	Correlation coefficients and a fitting curve were calculated and visualized. Higher PDC of statin use was associated with reduced OA risk (R = −0.91, *p* = 0.034) except for the >5-year subgroup (R = 0.51, *p* = 0.38). However, longer statin use follow-up duration may increase OA risk. Meta-analyzed estimations in the same persistence of >5-year vs. >1-year were significantly increased (OR 1.204 [95% CI 1.143–1.270, *p* < 0.001]).
Clockaerts et al. (2012)	Only data on correlations of OA progression and durations were available.	Statin use is associated with a more than 50% reduction in overall progression of OA of the knee.	The data of OA progression of <120-day, 120–364-day and >365-day durations of statin use were meta-analyzed and calculated by correlation analysis. Statin use may not be associated with decreased risk of OA progression (R = -0.82, *p* = 0.38)
Dosage cohort			
Kadam et al. (2013)	As above	As above	In overall cohorts, including nonusers, no significant correlation was detected (R = -0.57, *p* = 0.32), but from the lowest dose to the highest dose of statin use, a significant negative correlation was detected (R = -0.96, *p* = 0.038). A decreasing trend was found in the Cochran–Armitage trend test (Z = 4.2896, *p* < 0.001), indicating higher dose of statin use decreased OA risk.
Cheng et al. (2018)	No consensus estimation available to be meta-analyzed with overall cohorts.	Higher dose of statins reduced the incidence of spinal degenerative joint disease in patients with hypercholesterolemia.	The reference group of this study was the lowest dose (5400 mg total in observation period) of statin use, the outcomes revealed that the nonuser group had a significantly lower OA risk. If the estimations were transformed to nonusers as the reference group following the method of Hamling et al. (2008), statin use was linked to higher OA risk overall (OR 1.349 [95%CI 1.267–1.437]) and in each dose group. These analyses contradicted the conclusion of this study.
			In overall cohorts including nonusers, no significant correlation was detected (R = 0.16, *p* = 0.80). If the nonuser group was removed, OA risk decreased from lowest to highest dose of statin use (R = -0.97, *p* = 0.026). A negative trend was also observed in the Cochran–Armitage trend test (Z = 16.153, *p* < 0.001).
Chodick et al. (2010)	As above	As above	Due to lack of detail about the number of participants with OA in every dose level, we meta-analyzed the HRs of medium and high vs. low efficacy (References) in the 1- and 5-year follow-up groups. A higher dose of statin use was associated with increased OA risk in the 1-year group (OR 1.091 [95%CI 1.009–1.179, I^2^ = 0.00%, *p* = 0.029]), but not in the 5-year group (OR 1.004 [95%CI 0.886–1.138, I^2^ = 28.9%, *p* = 0.948]).
Surgery cohort			
Jonsson et al. (2019)	No other data were available to be analyzed together.	Statin use was not associated with total knee arthroplasty (TKA) due to OA in both male (*p* = 0.93) and female (*p* = 0.36) patients.	Statin use did not increase the risk of TKA due to OA, both in the AGES II period (*p* = 0.990) and in the 5-year incidence (*p* = 0.200) by meta-analysis.

For estimations concerning statin use and OA-related surgery, five candidate studies were selected where an OA-related surgery (total joint arthroplasty/revision/osteotomy) was defined as the endpoint event. Statin use was linked to a higher number of OA-related surgery in raw data (OR 1.216 [95% CI 1.190–1.242, *p* < 0.001]) but not in adjusted data.

### OA progression and statin use

Six studies examined OA progression. The relevant data were reported and analyzed in two ways: the follow-up endpoint vs. baseline (OR 1.034 [95% CI 0.798–1.341, *p* = 0.800]) and adjusted estimations (OR 0.867 [95% CI 0.557–1.349, *p* = 0.527]). No significant outcomes were detected in either analysis, indicating that statin use was not associated with OA progression or radiologic progression (Perry et al., 2021) (Table 3).

### Duration analysis

The links between statin use duration and OA risks were analyzed in nine studies. Significantly higher risks were only detected in cohorts of “former statin use” (OR 1.169 [95% CI 1.022–1.338, *p* = 0.023]), but the definition was not clear. To further estimate the continuous tendency of duration–risk correlations, Pearson’s correlation coefficients and fitting curves were calculated and visualized with the ggplot2 and ggpubr packages (Supplementary Figures S1A,B). Correlation coefficients of two measurements of tendency did not indicate a significant trend (R = −0.063, *p* = 0.89; R = 0.53, *p* = 0.28). Comparisons of the estimations were made every two adjacent durations, and meta-analyses of these ratios were performed. No significant outcomes were found, indicated that when other factors were consistent, statin use duration was not associated with OA risk.

The non-meta-analyzed data of two duration cohorts in Chodick et al. (2010) revealed that higher persistent statin use could decrease OA risk in the >1-year follow-up cohort when correlation coefficients were calculated and a significant negative correlation was observed (R = −0.91, *p* = 0.034). This negative correlation was not observed in the >5-year cohort (R = 0.51, *p* = 0.38). However, a higher risk of OA was seen in the >5-year cohort (OR 1.065 [95%CI 1.016–1.116, *p* < 0.001]), and meta-analyzed estimations in the same persistence of >5-year vs. >1-year was significantly increased (OR 1.204 [95% CI 1.143–1.270, *p* < 0.001]) (Table 3). No other significant outcome or tendency was found.

### Dose analysis

Dose analysis was tested in five studies. Based on the provided data and statin dose equivalent conversions (Weng et al., 2010), two subgroups were identified: 1) Statin users vs. nonusers, and 2) antihypertensive drugs (AHDs) with statin use vs. AHDs without statin use. For better visual and qualitative presentations regarding dose estimation correlations, the subgroups of these two groups were set as nonusers, low-dose (simvastatin daily <20 mg), low-medium (<40 mg), medium (40 mg), medium-high (40–80 mg), and high-dose (>80 mg). Medium-high (OR 1.079 [95% CI 1.012–1.151, *p* = 0.021]) and high doses (OR 1.383 [95% CI 1.054–1.815, *p* = 0.019]) of statin use were significantly associated with higher OA risks. A sudden increase in ORs was seen in the high-dose subgroup compared with the other dose groups, suggesting that a dose threshold could exist beyond which higher doses are associated with significantly higher OA risk. When 20 mg/d simvastatin equivalent was set as a threshold, overall >20 mg/d vs. <20 mg/d of statin use calculated by meta-analyzing the estimations of a higher dose vs. adjacent lower one, higher statin use was associated with increased OA risk (OR 1.108 [95% CI 1.004–1.222, *p* = 0.041]).

For the tendency analysis of dose-risk estimations from the low-dose to high-dose, positive trends that approached significance were detected (*p* = 0.8, R = 0.057) (Supplementary Figure S1C). However, based on the evidence obtained from meta-analyses of each adjacent higher dose vs. lower, higher statin doses slightly increased OA risk in the overall cohort (OR 1.039 [95% CI 1.000–1.080, *p* = 0.049]).

Cochran–Armitage tests for trends were performed using the DescTools package for non-meta-analyzed studies where binomial case and control numbers of every dose level were available. Two studies (Kadam et al., 2013; Cheng et al., 2018) indicated that OA risk was detected as being significantly reduced with higher statin dose, as the dose-HR correlation fell when the nonuser group was removed. Note, however, the nonuser group was not set as the reference in Cheng et al. (2018). If the nonuser group was transformed as the reference, statin use may significantly increase OA risk, regardless of dose level (Table 3). The evidence above indicates that, when other factors are consistent, higher statin doses may be linked to increased OA risk.

### AHDs use in statin users on the OA and related surgeries risk

Only Michaëlsson et al. (2017) analyzed the association of AHDs and OA-related outcomes of statin users. The effect of AHDs was only analyzed in sensitivity analyses without an explanation of why it was listed as a potential confounder factor. In this four-center study, we set two groups as group-1 and group-2 in available data. Group 1 included the estimations of participants with both AHD and statin use vs. AHD users only; meanwhile, group 2 was defined as overall statin users vs. nonusers, regardless of AHD use. Using the method of Altman Altman and Bland (2003), we calculated the estimations of group 1 vs. group 2 by corresponding study cohort, then these calculated estimations were meta-analyzed and defined as EST-1 (data from Table 2 of Michaëlsson et al., 2017). EST-2 was defined as group 1 vs. group 2 above, and estimations of every dose were used (data from Table 4 of Michaëlsson et al., 2017). In these comparisons, AHDs were considered a potential intervention. AHDs were associated with significantly decreased OA risk and OA-related surgeries in both EST-1 (OA risk (OR 0.901 [95% CI 0.825–0.984, *p* = 0.020]), surgery (OR 0.912 [95% CI 0.833–0.999, *p* = 0.048])) and EST-2 (OA risk (OR 0.906 [95% CI 0.837–0.981, *p* = 0.015]), surgery (OR 0.882 [95% CI 0.783–0.994, *p* = 0.039])), but these outcomes may be only available for hypertensive statin users (Figure 2C). These outcomes may be explained as follows: because comparisons involved whether to administer AHDs, the risks of OA and related surgeries were lower in the AHD group because of significant blood pressure control compared with those in the overall group, as hypertension was determined to be linked to a higher likelihood of OA development. Because group 2 included both hypertensive participants with and without AHD use, the impact of AHDs in decreasing OA-related outcomes may be inferred as more significant when the blood pressure is well-controlled in hypertensive statin users than when it is not well controlled. However, the existence of interactions between AHDs and statins remains unclear and needs further investigation.

### Heterogeneity and publication bias

All cohorts were subjected to heterogeneity testing, but significant heterogeneity was only detected in the raw data of the risk analyses. Subgroup analyses were only conducted based on both adjusted estimations and raw data of the OA-risk analyses, and outcomes were preferred when obtained from multiple studies. Statin use was detected as being significantly increased in a Caucasian population, in cohort study design, in longer follow-up durations and in small-joint OA like hand OA, suggesting that these situations of statin use need further consideration. No additional significant outcomes were detected, and the outcomes of the subgroup analysis are listed in Supplementary Table S3.

Sensitivity analyses were performed with no eliminations that could have been caused by multiple and heterogeneous methodologies. Meta-regression was thus performed for raw data analysis. The overall variables accounted for all heterogeneity (97.40%), and among those variables, race, lesion, follow-up periods and NOS scores accounted for 54.45%, 38.75%, 43.41% and 18.06%, respectively. Publication bias tests were only conducted in risk analysis, and bias was not found in Egger’s or Begg’s tests for any cohort.

## Discussion

Beyond using statins as first-line medications to treat atherosclerotic cardiovascular events and dyslipidemia, the anti-inflammatory and cartilage-protective effects have been reported by numerous studies. Our meta-analysis comprehensively investigates whether statin use leads to OA-related outcomes, and suggest that statin use may be associated with OA-related outcomes, especially at higher doses. In addition, AHDs significantly decrease OA and related surgeries risks in hypertensive statin users.

In the previous meta-analysis (Wang et al., 2020), methodologic confusion led to controversial conclusions regarding statin use and OA development. First, the retrieval and use of the literature did not follow PRISMA guidelines. For instance, a study reported as a letter by Valdes et al. (2014) was included, but letter type was excluded during their literature retrieval. More eligible studies were included in our study compared with that study. Secondly, inappropriate data uses and transformations were also present in that study; the estimation transformation method was not appropriate and had no basis for some estimations with the same baseline reference, and some data regarding other OA types were ignored. Lastly, duplicate data were potentially used. Thus, previous meta-analysis was deemed controversial, and we attempted to overcome these issues in this meta-analysis.

The overall outcomes indicated that statin use may slightly increase the risk of OA, which was inconsistent with the previous meta-analysis. Statins have been reported to protect cartilage, and views are presented. First, cholesterol and fat were shown to be positively correlated with OA severity (Ali et al., 2016). Statins decrease circulating and intra-chondrocyte cholesterol and fats, downregulate and inhibit adipo-related inflammatory cytokines, and upregulate cartilage-protective factors. Second, it has been reported that vascular pathologies, including arteriosclerosis, could contribute to OA (Liu et al., 2019). The anti-arteriosclerosis function of statins could, therefore, be an attractive therapeutic target for OA. Third, it is believed that metabolic syndrome (MetS) is closely correlated with OA, and statins are known to reduce inflammatory cytokines in MetS and related diseases (Tabrizi et al., 2019). Fourth, statins have the effect of preventing inflammatory cell infiltration (Akasaki et al., 2009), as they are anti-oxidative and anti-inflammatory, thus alleviating pain and cartilage degeneration, blocking the further progression of symptoms.

As statins are widely and long-termly used in older people, their potential contributions to musculoskeletal disorders deserve considerable attention. Some studies reported that statin use was linked to musculoskeletal conditions, including arthropathies (Mansi et al., 2013). Statins have also been reported linked to commonly occurring myopathies with statin-associated muscle symptoms (SAMS) (Bouitbir et al., 2020). Thus, statin-related adverse effects, such aspotential weakening of musculoskeletal mechanical properties (Eliasson et al., 2019) and some metabolic interferences like diabetes mellitus (DM) may lead to OA. Based on this evidence and our outcomes, we hypothesized that statins increase OA-related outcome risks through musculoskeletal and metabolic interference pathways. Musculoskeletal disorders and associated symptoms induced by statins could lead to greater mechanical loads and reduced physical activities. However, whether statin use leads to decreased muscle and tendon function has remained controversial. In the subgroup analysis, more significant likelihood was found in hand OA than large-joint OA, and flexible joints were more likely to be interfered with by statins, which inferred that apart from mechanical loads, extensive musculoskeletal metabolic disturbance may exist due to statin use. Another potential mechanism is that a high percentage of older people with multiple comorbidities are included. They are more likely to develop MetS and sarcopenia, and it is possible that beneficial effects of statins are limited. Thus, detailed comorbidities of participants’ baseline conditions are needed to confirm or reject the presence of MetS or sacropenia. However, it is still important to highlight that attention should be paid to potential OA risks in long-term statin users, especially older people.

For statin use and OA-related outcomes, people taking higher statin doses are more likely to develop OA. High-dose statin use may increase SAMS development, and simvastatin >40 mg/d equivalents was associated with higher osteoporosis and diabetes risks (Swerdlow et al., 2015; Leutner et al., 2019). The statin use durations were detected as not associated with higher OA risk, but short-term statin use was reported to lead to musculoskeletal diseases, including OA, in subgroup analysis (Mansi et al., 2016). AHDs were detected as associated with lower OA and related surgeries risks in statin users, but this may not indicate that there must be interactions between AHDs and statins. Michaëlsson et al. (2017) took AHDs as the basis for the sensitivity analysis in their study, but the reason was not mentioned. AHDs were analyzed due to considerable data available for researching potential correlations. Hypertension was reported to be closely linked to OA, but the beneficial effects of AHDs in decreasing OA development risks and potential interactions with statins were inconclusive. Thus, these outcomes require additional studies for confirmation.

This study was performed following recommendations for rigorous meta-analyses. A major strength was the inclusion of large sample size comprising multiple population-based studies performed under rigorous research conditions or from databases based on credible records. This process provided an advantage by eliminating biases. Therefore, we feel that our meta-analysis outcomes are robust and valuable for further investigations, especially in patients with MetS. In our study, multiple statin use analyses were performed using major parameters, such as duration, doses, and multiple OA-related outcomes. We also used tendency analyses for dynamic estimations.

Although several challenges were encountered and resolved, there were also several significant limitations. First, as a limitation of drug-related meta-analysis of multiple retrospective large-sample studies, it is premature to arrive at an absolute causality. Heterogeneous baseline conditions, and confounding factors and methodologies were present, and heterogeneity could not be completely eliminated. Therefore, the conclusion was limited and challenged by inconsistent quality and multiple confounders in the included studies, let alone the lack of a rigorous causal relationship. Second, some important definitions of statin use were lacking or controversial. For example, statin prescriptions did not necessarily qualify as use or even regular daily use, and no consistent definition of statin use was present. However, as OA was a long-term progressive degenerative disease, logically and theoretically, it may be inferred that OA progress, incident and surgery risk are significantly elevated with long-duration and/or high-dose use when statins were deemed associated with OA. Multi-dimensional analysis was only performed in one included study that used total statin dosage taken within a certain duration (Cheng et al., 2018). Statin use duration and dose cannot be investigated simultaneously with the present available data. Third, the included studies should have had more detailed data, like dyslipidemia conditions, body mass index (BMI) and work intensity of participants, and data needed for transformation. Especially subgroup analysis of various types of statins could not be conducted due to lack of detailed information about the types of statins taken by the patients. In addition, the limited number of studies resulted in limited power for estimations in some analyses. We hope to eliminate heterogeneity and potential bias by including studies with confounding factors adjusted and with large number of participants. We hope that future rigorous studies could help to analyze each parameter of statin use and OA-related outcomes in more detail.

In this meta-analysis based on multiple population-based studies, the statistical outcomes inferred that statin use might be associated with increased OA development risk, especially at higher doses. These findings do not support the argument that statins alleviate OA risks and highlighted the importance of recognizing potential OA risk in the population with long-term and/or high-dose statin use, especially in an older population. In addition, AHDs reduced OA incident risk and OA-related surgeries in hypertensive statin users. However, because the methodologies and parameters of the included studies were various and heterogenous, more rigorous, multi-dimension studies with greater numbers of participants are needed to confirm our conclusions.

## Data Availability

The original contributions presented in the study are included in the article/Supplementary Material; further inquiries can be directed to the corresponding author.

## Supplementary material

Supplementary Figure S1 Correlation analysis of statin use duration and dosage and OA risk. **(A,B)** display two kinds of duration descriptions of statin use. The horizontal coordinate indicates statin use duration, and the ordinate coordinate indicates the OR values and 95% CIs of statin users vs. nonusers. Statin use dose-OA risk correlation analysis of **(C)** overall statin users and **(D)** statin users with antihypertensive drug use. The horizontal coordinate here indicates statin use dosage. No significant correlations and tendencies were detected.
