# Supplementary material for: The association between statin use and osteoarthritis-related outcomes: An updated systematic review and meta-analysis
Source: Front Pharmacol. 2022 Nov 24;13:1003370. doi: 10.3389/fphar.2022.1003370 (PMC9729269; doi:10.3389/fphar.2022.1003370)
Supplement: Supplementary file 1 [file Table1.docx]

**Supplementary Table S1. Data sources and potentially duplicate data**

**A. Potentially duplicate data**

| **Study** | **Involved database** | **Preferred study or data** | **Reason** |
| --- | --- | --- | --- |
| Riddle et al (2012) [38] | OAI | Risk cohort: Veronese et al (2018) [17]; Dosage and duration cohort: Riddle et al (2012) [38]; Progression cohort: Veronese et al (2018) [17]. | The study of Veronese et al (2018) [17] included the largest number of participants of OAI. And in the study of Riddle et al (2012) [38], detailed duration-subject numbers were available. |
| Veronese et al (2018) [17] |  |  |  |
| Haj-Mirzaian et al (2019) [18] |  |  |  |
| Burkard et al (2018) [41] | CPRD/GPRD | Risk cohort: number of participants and HR values: Burkard et al (2018) [41], OR value: Frey et al (2017) [34]. Surgery cohort: adjusted estimations: Sarmanova et al (2020) [48]; crude data: Cook et al (2019) [45]. | The CPRD study was previously known as GPRD. The study of Burkard et al (2018) [41] had more PSM participants and HR values. OR value was only available in the study of Frey, et al. Largest PSMed data of arthroplasty were only available in the study of Sarmanova et al (2020) [48], but largest crude data were only available in Cook et al (2019) [45]. |
| Frey et al (2017) [34] |  |  |  |
| Cook et al (2019) [45] |  |  |  |
| Kadam et al (2013) [47] |  |  |  |
| Sarmanova et al (2020) [48] |  |  |  |

**B. Sources of data in included studies**

| **Author** | **Year** | **Country** | **Meta-analysis** | **Systematic review** | **Data source** |
| --- | --- | --- | --- | --- | --- |
| Frey et al (2017) [34] | 2017 | Switzerland | NA | Risk | Table 3 |
| Eymard et al (2018) [35] | 2018 | France | Progression | Progression | Table 1-2 |
| Valdes et al (2014) [15] | 2014 | UK | Risk | Risk | Table 1-2 |
| Beattie et al (2005) [43] | 2005 | USA | Risk and progression | Risk and progression | Table 1-3 |
| Jonsson et al (2019) [36] | 2019 | Iceland | Risk | Risk | Table 1 |
| Chodick et al (2010) [37] | 2010 | Israel | Risk, dosage and duration | Risk, dosage and duration | Table 2, 3, 5 |
| Riddle et al (2012) [38] | 2012 | USA | NA | Risk and duration | Table 1 |
| Kadam et al (2013) [47] | 2013 | UK | Risk, dosage and duration | Risk, dosage and duration | Table 2 |
| Cheng et al (2018) [39] | 2018 | China | Risk and dosage | Risk and dosage | Table 1 |
| Garcia-Gil et al (2017) [40] | 2017 | Spain | Risk | Risk | Table 2 |
| Veronese et al (2018) [17] | 2018 | UK | Risk and duration | Risk and duration | Table 1-3 |
| Burkard et al (2018) [41] | 2018 | Switzerland | Risk, dosage and duration | Risk, dosage and duration | Table 2 and Supplementary Table 4-5 |
| Roy et al (2017) [42] | 2017 | USA | Risk | Risk | Table 2 |
| Clockaerts et al (2012) [16] | 2012 | Netherlands | Risk, progression and duration | Risk, progression and duration | Table 2-3 |
| Michaelsson et al (2017) [19] | 2017 | Sweden | Risk, dosage, surgery and duration | Risk, dosage, surgery and duration | Table 2-4 |
| Haj-Mirzaian et al (2019) [18] | 2019 | USA | Duration and progression | Duration, risk and progression | Table 2-4 |
| Cemeroglu et al (2014) [44] | 2014 | Turkey | Risk | Risk | Table 1 |
| Cook et al (2019) [45] | 2019 | UK | NA | Surgery | Table 1-2 |
| Chaganti et al (2012) [46] | 2012 | USA | Risk | Risk | Table |
| Sarmanova et al (2020) [48] | 2020 | UK | Surgery | NA | Table 3 |
| Perry et al (2021) [33] | 2021 | UK | NA | Progression | Table 2 |
| Simic et al (2021) [32] | 2021 | Australia | Progression | NA | Table 2 |
| Mohajer et al (2021) [49] | 2022 | USA | NA | Risk | Table 2 |
| Mansi et al (2013) [12] | 2013 | USA | Risk | Risk | Table 1 |
