# Supplementary material for: The association between statin use and osteoarthritis-related outcomes: An updated systematic review and meta-analysis
Source: Front Pharmacol. 2022 Nov 24;13:1003370. doi: 10.3389/fphar.2022.1003370 (PMC9729269; doi:10.3389/fphar.2022.1003370)
Supplement: Supplementary file 2 [file Presentation1.pdf]

Database: PubMed

(osteoarthriti[All Fields] OR osteoarthritic[All Fields] OR osteoarthritic'[All Fields] OR osteoarthritics[All Fields] OR osteoarthritides[All Fields] OR osteoarthrities[All Fields] OR osteoarthritis[All Fields] OR osteoarthritis'[All Fields] OR osteoarthritisand[All Fields] OR osteoarthritisassociated[All Fields] OR osteoarthritisderived[All Fields] OR osteoarthritisis[All Fields] OR osteoarthritislike[All Fields] OR osteoarthritisprogression[All Fields] OR osteoarthritisrabbit[All Fields] OR osteoarthritiss[All Fields] OR osteoarthritistis[All Fields] OR osteoarthrititis[All Fields] OR osteoarthritits[All Fields]) OR (("osteoarthritis"[MeSH Terms] OR "osteoarthritis"[All Fields]) OR (osteoarthritc[All Fields] OR osteoarthrite[All Fields] OR osteoarthrites[All Fields] OR osteoarthrith[All Fields] OR osteoarthrithis[All Fields] OR osteoarthriti[All Fields] OR osteoarthritic[All Fields] OR osteoarthritic'[All Fields] OR osteoarthritics[All Fields] OR osteoarthritides[All Fields] OR osteoarthrities[All Fields] OR osteoarthritis[All Fields] OR osteoarthritis'[All Fields] OR osteoarthritisand[All Fields] OR osteoarthritisassociated[All Fields] OR osteoarthritisderived[All Fields] OR osteoarthritisis[All Fields] OR osteoarthritislike[All Fields] OR osteoarthritisprogression[All Fields] OR osteoarthritisrabbit[All Fields] OR osteoarthritiss[All Fields] OR osteoarthritistis[All Fields] OR osteoarthrititis[All Fields] OR osteoarthritits[All Fields] OR osteoarthrits[All Fields]) OR (osteo arthrite[All Fields] OR osteo arthrites[All Fields] OR osteo arthritic[All Fields] OR osteo arthritics[All Fields] OR osteo arthritis[All Fields]) OR (osteoarthro[All Fields] OR osteoarthroapthy[All Fields] OR osteoarthrocutaneous[All Fields] OR osteoarthrodermopathic[All Fields] OR osteoarthrodesis[All Fields] OR osteoarthrodysplasia[All Fields] OR osteoarthrography[All Fields] OR osteoarthrologiai[All Fields] OR osteoarthrological[All Fields] OR osteoarthrology[All Fields] OR osteoarthromuscular[All Fields] OR osteoarthromusculovascular[All Fields] OR osteoarthromyalgias[All Fields] OR osteoarthromyopathies[All Fields] OR osteoarthroonychodysplasie[All Fields] OR osteoarthropathe[All Fields] OR osteoarthropathi[All Fields] OR osteoarthropathi'aja[All Fields] OR osteoarthropathia[All Fields] OR osteoarthropathiaja[All Fields] OR osteoarthropathic[All Fields] OR osteoarthropathie[All Fields] OR osteoarthropathien[All Fields] OR osteoarthropathies[All Fields] OR osteoarthropathique[All Fields] OR osteoarthropathy[All Fields] OR osteoarthropathy1[All Fields] OR osteoarthropatia[All Fields] OR osteoarthropatias[All Fields] OR osteoarthropatic[All Fields] OR osteoarthropatie[All Fields] OR osteoarthropaties[All Fields] OR osteoarthrophy[All Fields] OR osteoarthrophytes[All Fields] OR osteoarthroplasty[All Fields] OR osteoarthros[All Fields] OR osteoarthrose[All Fields] OR osteoarthrosed[All Fields] OR osteoarthroseentwicklung[All Fields] OR osteoarthrosen[All Fields] OR osteoarthroser[All Fields] OR osteoarthroses[All Fields] OR osteoarthrosic[All Fields] OR osteoarthrosique[All Fields] OR osteoarthrosis[All Fields] OR osteoarthrosis'[All Fields] OR osteoarthrosis,[All Fields] OR

osteoarthrosisok[All Fields] OR osteoarthrositis[All Fields] OR osteoarthrotendinous[All Fields] OR osteoarthrotenocutaneous[All Fields] OR osteoarthrotic[All Fields] OR osteoarthrotically[All Fields] OR osteoarthrotics[All Fields] OR osteoarthrotis[All Fields] OR osteoarthrotischen[All Fields] OR osteoarthrotischer[All Fields] OR osteoarthrotomy[All Fields]) OR (osteo arthrodesis[All Fields] OR osteo arthrodysplasia[All Fields] OR osteo arthropathia[All Fields] OR osteo arthropathic[All Fields] OR osteo arthropathie[All Fields] OR osteo arthropathies[All Fields] OR osteo arthropathique[All Fields] OR osteo arthropathy[All Fields] OR osteo arthropie[All Fields] OR osteo arthroplastic[All Fields] OR osteo arthrose[All Fields] OR osteo arthroses[All Fields] OR osteo arthrosic[All Fields] OR osteo arthrosis[All Fields] OR osteo arthrotic[All Fields]) OR (osteoarthro[All Fields] OR osteoarthroapthy[All Fields] OR osteoarthrocutaneous[All Fields] OR osteoarthrodermopathic[All Fields] OR osteoarthrodesis[All Fields] OR osteoarthrodysplasia[All Fields] OR osteoarthrography[All Fields] OR osteoarthrologiai[All Fields] OR osteoarthrological[All Fields] OR osteoarthrology[All Fields] OR osteoarthromuscular[All Fields] OR osteoarthromusculovascular[All Fields] OR osteoarthromyalgias[All Fields] OR osteoarthromyopathies[All Fields] OR osteoarthroonychodysplasie[All Fields] OR osteoarthropathe[All Fields] OR osteoarthropathi[All Fields] OR osteoarthropathi'aja[All Fields] OR osteoarthropathia[All Fields] OR osteoarthropathiaja[All Fields] OR osteoarthropathic[All Fields] OR osteoarthropathie[All Fields] OR osteoarthropathien[All Fields] OR osteoarthropathies[All Fields] OR osteoarthropathique[All Fields] OR osteoarthropathy[All Fields] OR osteoarthropathy1[All Fields] OR osteoarthropatia[All Fields] OR osteoarthropatias[All Fields] OR osteoarthropatic[All Fields] OR osteoarthropatie[All Fields] OR osteoarthropaties[All Fields] OR osteoarthrophy[All Fields] OR osteoarthrophytes[All Fields] OR osteoarthroplasty[All Fields] OR osteoarthros[All Fields] OR osteoarthrose[All Fields] OR osteoarthrosed[All Fields] OR osteoarthroseentwicklung[All Fields] OR osteoarthrosen[All Fields] OR osteoarthroser[All Fields] OR osteoarthroses[All Fields] OR osteoarthrosic[All Fields] OR osteoarthrosique[All Fields] OR osteoarthrosis[All Fields] OR osteoarthrosis'[All Fields] OR osteoarthrosis,[All Fields] OR osteoarthrosisok[All Fields] OR osteoarthrositis[All Fields] OR osteoarthrotendinous[All Fields] OR osteoarthrotenocutaneous[All Fields] OR osteoarthrotic[All Fields] OR osteoarthrotically[All Fields] OR osteoarthrotics[All Fields] OR osteoarthrotis[All Fields] OR osteoarthrotischen[All Fields] OR osteoarthrotischer[All Fields] OR osteoarthrotomy[All Fields]) OR (gonarthritic[All Fields] OR gonarthritiden[All Fields] OR gonarthritides[All Fields] OR gonarthritis[All Fields]) OR (gonarthrocace[All Fields] OR gonarthrographe[All Fields] OR gonarthroiis[All Fields] OR gonarthromeningitis[All Fields] OR gonarthromuscular[All Fields] OR gonarthromuskulares[All Fields] OR gonarthropathies[All Fields] OR gonarthropathy[All Fields] OR gonarthroplasty[All Fields] OR gonarthros[All Fields] OR gonarthroscopy[All Fields] OR gonarthrose[All Fields] OR

gonarthrosebehandlung[All Fields] OR gonarthrosen[All Fields] OR gonarthrosepatienten[All Fields] OR gonarthroseprogedienz[All Fields] OR gonarthroses[All Fields] OR gonarthrosetherapie[All Fields] OR gonarthrosic[All Fields] OR gonarthrosique[All Fields] OR gonarthrosiques[All Fields] OR gonarthrosis[All Fields] OR gonarthrosis'[All Fields] OR gonarthrotic[All Fields] OR gonarthroze[All Fields]) OR (coxarthritic[All Fields] OR coxarthritis[All Fields] OR coxarthritis"[All Fields]) OR (coxarthro[All Fields] OR coxarthrodesis[All Fields] OR coxarthrogenic[All Fields] OR coxarthrography[All Fields] OR coxarthropathies[All Fields] OR coxarthropathy[All Fields] OR coxarthroplasty[All Fields] OR coxarthros[All Fields] OR coxarthrose[All Fields] OR coxarthrosebehandlung[All Fields] OR coxarthrosebehandlungen[All Fields] OR coxarthrosefragen[All Fields] OR coxarthrosen[All Fields] OR coxarthroseproblem[All Fields] OR coxarthroses[All Fields] OR coxarthrosezeichen[All Fields] OR coxarthrosic[All Fields] OR coxarthrosique[All Fields] OR coxarthrosiques[All Fields] OR coxarthrosis[All Fields] OR coxarthrossi[All Fields] OR coxarthrotic[All Fields] OR coxarthroxis[All Fields] OR coxarthroz[All Fields]) OR (arthros[All Fields] OR arthrosa[All Fields] OR arthrosas[All Fields] OR arthrosaura[All Fields] OR arthrosc[All Fields] OR arthroscan[All Fields] OR arthroscanner[All Fields] OR arthroscanners[All Fields] OR arthroscanning[All Fields] OR arthroscanography[All Fields] OR arthroscans[All Fields] OR arthroscentesis[All Fields] OR arthroscintigram[All Fields] OR arthroscintigraphic[All Fields] OR arthroscintigraphy[All Fields] OR arthroscintography[All Fields] OR arthroscipique[All Fields] OR arthrosclerose[All Fields] OR arthrosclerosis[All Fields] OR arthrosclerotic[All Fields] OR arthrosco[All Fields] OR arthroscop[All Fields] OR arthroscopal[All Fields] OR arthroscope[All Fields] OR arthroscope's[All Fields] OR arthroscoped[All Fields] OR arthroscopes[All Fields] OR arthroscopi[All Fields] OR arthroscopia[All Fields] OR arthroscopially[All Fields] OR arthroscopiaval[All Fields] OR arthroscopic[All Fields] OR arthroscopic'[All Fields] OR arthroscopicacromioplasty[All Fields] OR arthroscopical[All Fields] OR arthroscopically[All Fields] OR arthroscopicassisted[All Fields] OR arthroscopiclly[All Fields] OR arthroscopicmeniscectomy[All Fields] OR arthroscopico[All Fields] OR arthroscopics[All Fields] OR arthroscopie[All Fields] OR arthroscopied[All Fields] OR arthroscopies[All Fields] OR arthroscopilally[All Fields] OR arthroscopique[All Fields] OR arthroscopiques[All Fields] OR arthroscopis[All Fields] OR arthroscopist[All Fields] OR arthroscopist's[All Fields] OR arthroscopists[All Fields] OR arthroscopists'[All Fields] OR arthroscopoic[All Fields] OR arthroscopos[All Fields] OR arthroscopy[All Fields] OR arthroscopy'[All Fields] OR arthroscopy's[All Fields] OR arthroscopy1[All Fields] OR arthroscopy13[All Fields] OR arthroscopy2001[All Fields] OR arthroscopya[All Fields] OR arthroscopyando[All Fields] OR arthroscopyassisted[All Fields] OR arthroscopytechniques[All Fields] OR arthroscpec[All Fields] OR arthroscpic[All Fields] OR arthroscpoe[All Fields] OR arthroscpoic[All Fields] OR arthrose[All Fields] OR arthrose1[All Fields] OR arthrose1,2[All Fields] OR arthroseacetabula[All Fields] OR arthroseassoziierte[All Fields] OR arthroseauslosende[All Fields] OR arthroseauslosung[All Fields] OR

arthrosebedingte[All Fields] OR arthrosebehandeling[All Fields] OR arthrosebehandlung[All Fields] OR arthrosebeschwerden[All Fields] OR arthrosediagnostik[All Fields] OR arthroseentstehung[All Fields] OR arthroseentwicklung[All Fields] OR arthroseforschung[All Fields] OR arthroseforschungsverbundes[All Fields] OR arthrosegefahrdung[All Fields] OR arthrosegelenks[All Fields] OR arthrosegrads[All Fields] OR arthrosehaufigkeit[All Fields] OR arthrosehilfe[All Fields] OR arthroseindex[All Fields] OR arthroseinduktion[All Fields] OR arthroseknie[All Fields] OR arthrosekniegelenke[All Fields] OR arthroseknorpel[All Fields] OR arthrosekranken[All Fields] OR arthrosekrankheit[All Fields] OR arthrosemerkmalen[All Fields] OR arthrosemodell[All Fields] OR arthrosen[All Fields] OR arthrosenbehandlung[All Fields] OR arthrosenbildung[All Fields] OR arthrosendiagnostik[All Fields] OR arthrosenepidemiologie[All Fields] OR arthrosenex[All Fields] OR arthrosenpathogenese[All Fields] OR arthrosentherapie[All Fields] OR arthroseopy[All Fields] OR arthroseos[All Fields] OR arthrosepatienten[All Fields] OR arthrosepatienter[All Fields] OR arthrosepatientin[All Fields] OR arthroseproblem[All Fields] OR arthroseprogression[All Fields] OR arthroseprophylaktischen[All Fields] OR arthroseprophylaxe[All Fields] OR arthroseprozess[All Fields] OR arthroseptatienten[All Fields] OR arthroser[All Fields] OR arthroseries[All Fields] OR arthroserisiko[All Fields] OR arthroses[All Fields] OR arthroseschmerz[All Fields] OR arthroseschmerzen[All Fields] OR arthroseschmerzes[All Fields] OR arthrosetherapie[All Fields] OR arthrosetten[All Fields] OR arthroseursache[All Fields] OR arthrosew[All Fields] OR arthrosezeichen[All Fields] OR arthrosi[All Fields] OR arthrosia[All Fields] OR arthrosic[All Fields] OR arthrosico[All Fields] OR arthrosics[All Fields] OR arthroside[All Fields] OR arthrosides[All Fields] OR arthrosies[All Fields] OR arthrosim[All Fields] OR arthrosinovitis[All Fields] OR arthrosique[All Fields] OR arthrosiques[All Fields] OR arthrosis[All Fields] OR arthrosis'[All Fields] OR arthrosisaund[All Fields] OR arthrosisban[All Fields] OR arthrosisra[All Fields] OR arthrositic[All Fields] OR arthrositis[All Fields] OR arthroskipische[All Fields] OR arthroskop[All Fields] OR arthroskope[All Fields] OR arthroskopias[All Fields] OR arthroskopiche[All Fields] OR arthroskopie[All Fields] OR arthroskopiebefund[All Fields] OR arthroskopiebefunde[All Fields] OR arthroskopien[All Fields] OR arthroskopietagung[All Fields] OR arthroskopisch[All Fields] OR arthroskopische[All Fields] OR arthroskopischem[All Fields] OR arthroskopischen[All Fields] OR arthroskopischer[All Fields] OR arthroskopisches[All Fields] OR arthroskopisher[All Fields] OR arthroso[All Fields] OR arthrosoarthritis[All Fields] OR arthrosocopic[All Fields] OR arthrosocopy[All Fields] OR arthrosocpy[All Fields] OR arthrosonografie[All Fields] OR arthrosonografische[All Fields] OR arthrosonogram[All Fields] OR arthrosonographic[All Fields] OR arthrosonographical[All Fields] OR arthrosonographically[All Fields] OR arthrosonographie[All Fields] OR arthrosonography[All Fields] OR arthrosopic[All Fields] OR arthrosopically[All Fields] OR arthrosopy[All Fields] OR arthrospec[All Fields] OR arthrosphaera[All

Fields] OR arthrosphaerae[All Fields] OR arthrosphaeridae[All Fields] OR arthrosphores[All Fields] OR arthrospine[All Fields] OR arthrospira[All Fields] OR arthrospirai[All Fields] OR arthrospiraplatensis[All Fields] OR arthrosplasty[All Fields] OR arthrospora[All Fields] OR arthrospore[All Fields] OR arthrospores[All Fields] OR arthrosporic[All Fields] OR arthrosporiella[All Fields] OR arthrosporioides[All Fields] OR arthrosporium[All Fields] OR arthrosporogenesis[All Fields] OR arthrosporol[All Fields] OR arthrosporols[All Fields] OR arthrosporone[All Fields] OR arthrosporous[All Fields] OR arthrosport[All Fields] OR arthrosporulating[All Fields] OR arthrosporulation[All Fields] OR arthrostasis[All Fields] OR arthrosteal[All Fields] OR arthrosteitis[All Fields] OR arthrostema[All Fields] OR arthrosteopedics[All Fields] OR arthrostic[All Fields] OR arthrostigma[All Fields] OR arthrostoma[All Fields] OR arthrostomy[All Fields] OR arthrostrade[All Fields] OR arthrostress[All Fields] OR arthrostyla[All Fields] OR arthrostylidiinae[All Fields] OR arthrostylidioid[All Fields] OR arthrosurface[All Fields] OR arthrosurgery[All Fields] OR arthrosy[All Fields] OR arthrosynovial[All Fields] OR arthrosynoviaux[All Fields] OR arthrosynovitic[All Fields] OR arthrosynovitis[All Fields] OR arthrosyntheses[All Fields] OR arthrosynthesis[All Fields] OR arthroszintigrafie[All Fields] OR arthroszkopiaja[All Fields]) OR (arthrotammy[All Fields] OR arthrotardigrada[All Fields] OR arthrotardigrade[All Fields] OR arthrotardigrades[All Fields] OR arthrotec[All Fields] OR arthrotec's[All Fields] OR arthrotek[All Fields] OR arthrotenodesis[All Fields] OR arthrotenolyse[All Fields] OR arthrotenolysis[All Fields] OR arthroteq[All Fields] OR arthrotera[All Fields] OR arthroteras[All Fields] OR arthroteres[All Fields] OR arthroteros[All Fields] OR arthrotest[All Fields] OR arthrotetraose[All Fields] OR arthrothamnaceae[All Fields] OR arthrothamnus[All Fields] OR arthrotic[All Fields] OR arthrotical[All Fields] OR arthrotically[All Fields] OR arthroticum[All Fields] OR arthrotiker[All Fields] OR arthrotisch[All Fields] OR arthrotische[All Fields] OR arthrotischem[All Fields] OR arthrotischen[All Fields] OR arthrotischer[All Fields] OR arthrotmeterrel[All Fields] OR arthrotome[All Fields] OR arthrotomein[All Fields] OR arthrotomi[All Fields] OR arthrotomia[All Fields] OR arthrotomic[All Fields] OR arthrotomie[All Fields] OR arthrotomies[All Fields] OR arthrotomii[All Fields] OR arthrotomised[All Fields] OR arthrotomized[All Fields] OR arthrotomodensitometrique[All Fields] OR arthrotomodensitometry[All Fields] OR arthrotomogram[All Fields] OR arthrotomogrames[All Fields] OR arthrotomograms[All Fields] OR arthrotomographic[All Fields] OR arthrotomographically[All Fields] OR arthrotomographie[All Fields] OR arthrotomographies[All Fields] OR arthrotomographique[All Fields] OR arthrotomographs[All Fields] OR arthrotomography[All Fields] OR arthrotomolgraphy[All Fields] OR arthrotomty[All Fields] OR arthrotomy[All Fields] OR arthrotoxicity[All Fields] OR arthrotriaosylceramide[All Fields] OR arthrotrichus[All Fields] OR arthrotripsometer[All Fields] OR arthrotrophische[All Fields] OR arthrotropic[All Fields] OR arthrotropique[All Fields] OR arthrotropism[All Fields] OR arthrotropy[All Fields] OR arthrotunneler[All Fields] OR arthrotus[All Fields]) OR ("degenerative"[All Fields] AND "joint"[All Fields]

AND "disease"[All Fields]) OR "degenerative joint disease"[All Fields]) AND (("hydroxymethylglutaryl-coa reductase inhibitors"[Pharmacological Action] OR "hydroxymethylglutaryl-coa reductase inhibitors"[MeSH Terms] OR ("hydroxymethylglutaryl-coa"[All Fields] AND "reductase"[All Fields] AND "inhibitors"[All Fields]) OR "hydroxymethylglutaryl-coa reductase inhibitors"[All Fields] OR "statin"[All Fields]) OR ("hydroxymethylglutaryl-coa reductase inhibitors"[Pharmacological Action] OR "hydroxymethylglutaryl-coa reductase inhibitors"[MeSH Terms] OR ("hydroxymethylglutaryl-coa"[All Fields] AND "reductase"[All Fields] AND "inhibitors"[All Fields]) OR "hydroxymethylglutaryl-coa reductase inhibitors"[All Fields] OR "statin"[All Fields]) OR *statin OR ("atorvastatin"[MeSH Terms] OR "atorvastatin"[All Fields]) OR ("cerivastatin"[Supplementary Concept] OR "cerivastatin"[All Fields]) OR ("crilvastatin"[Supplementary Concept] OR "crilvastatin"[All Fields]) OR ("lovastatin"[MeSH Terms] OR "lovastatin"[All Fields]) OR ("mevastatin"[Supplementary Concept] OR "mevastatin"[All Fields]) OR ("pitavastatin"[Supplementary Concept] OR "pitavastatin"[All Fields]) OR ("pravastatin"[MeSH Terms] OR "pravastatin"[All Fields]) OR ("rosuvastatin calcium"[MeSH Terms] OR ("rosuvastatin"[All Fields] AND "calcium"[All Fields]) OR "rosuvastatin calcium"[All Fields] OR "rosuvastatin"[All Fields]) OR ("simvastatin"[MeSH Terms] OR "simvastatin"[All Fields]))

Cochrane library

(Statin OR *statin OR Atorvastatin OR cerivastatin OR crilvastatin OR Lovastatin OR mevastatin OR pitavastatin OR Pravastatin OR Rosuvastatin OR Simvastatin) AND (Osteoarthrit* OR osteo-arthrit* OR osteoarthro* OR osteo-arthro* OR “degenerative arthritis” OR osteoarthritis)

Embase

('statin'/exp OR statin OR 'atorvastatin'/exp OR atorvastatin OR 'cerivastatin'/exp OR cerivastatin OR 'crilvastatin'/exp OR crilvastatin OR 'lovastatin'/exp OR lovastatin OR 'mevastatin'/exp OR mevastatin OR 'pitavastatin'/exp OR pitavastatin OR 'pravastatin'/exp OR pravastatin OR 'rosuvastatin'/exp OR rosuvastatin OR 'simvastatin'/exp OR simvastatin) AND ('osteoarthritis'/exp OR osteoarthritis)

Web of Science

((Statin OR *statin OR Atorvastatin OR cerivastatin OR crilvastatin OR Lovastatin OR mevastatin OR pitavastatin OR Pravastatin OR Rosuvastatin OR Simvastatin) AND (Osteoarthrit* OR osteo-arthrit* OR osteoarthro* OR osteo-arthro* OR “degenerative arthritis” OR osteoarthritis))

Scopus

((Statin OR *statin OR Atorvastatin OR cerivastatin OR crilvastatin OR Lovastatin OR mevastatin OR pitavastatin OR Pravastatin OR Rosuvastatin OR Simvastatin) AND (Osteoarthrit* OR osteoarthro* OR “degenerative arthritis” OR osteoarthritis))
