# Supplementary figures and images for: The association between statin use and osteoarthritis-related outcomes: An updated systematic review and meta-analysis
Source: Front Pharmacol. 2022 Nov 24;13:1003370. doi: 10.3389/fphar.2022.1003370 (PMC9729269; doi:10.3389/fphar.2022.1003370)

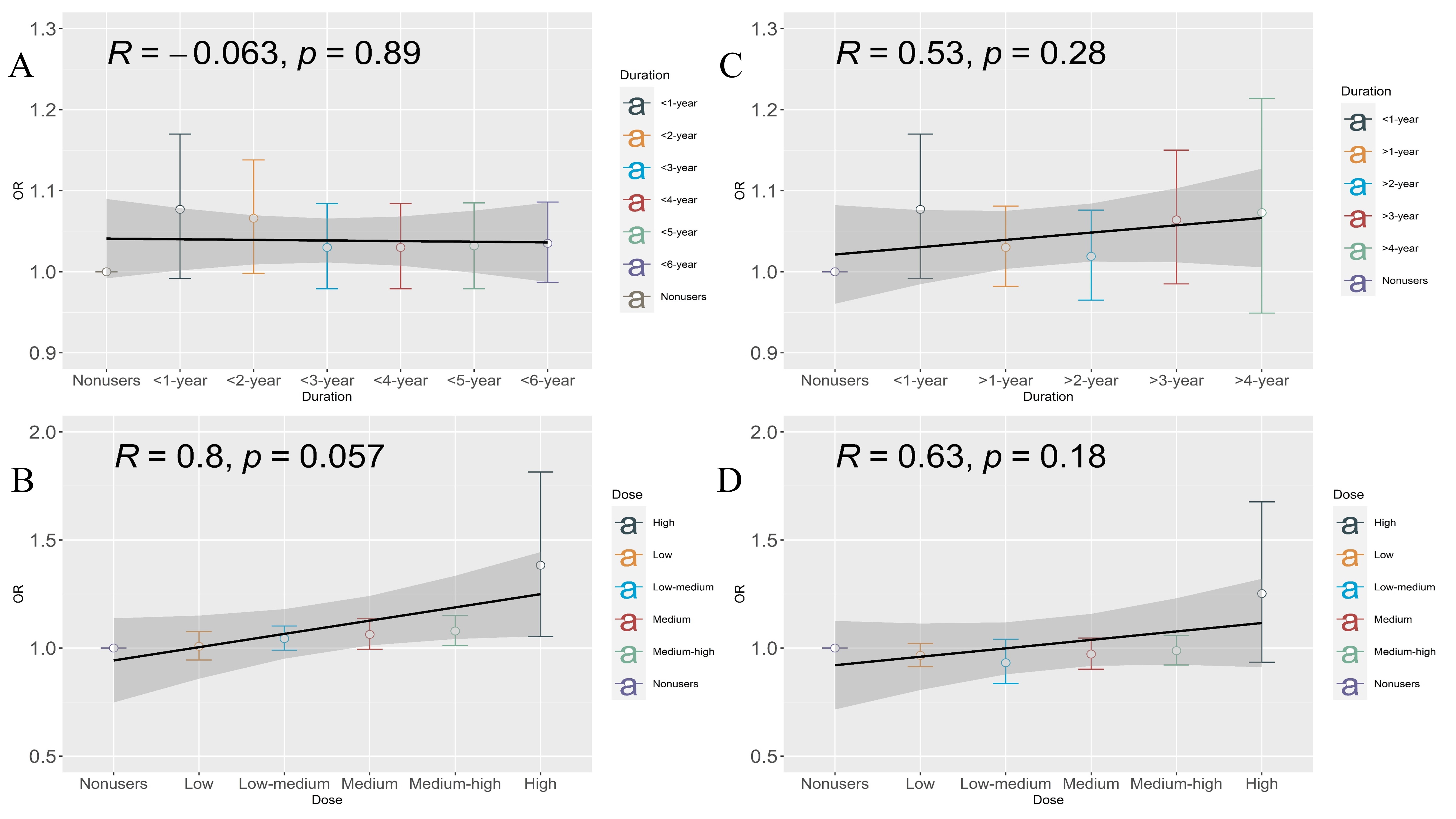

Supplement: Supplementary file 3 [file Image1.JPEG]
