# Supplementary material for: The association between statin use and osteoarthritis-related outcomes: An updated systematic review and meta-analysis
Source: Front Pharmacol. 2022 Nov 24;13:1003370. doi: 10.3389/fphar.2022.1003370 (PMC9729269; doi:10.3389/fphar.2022.1003370)
Supplement: Supplementary file 5 [file Table3.docx]

**Supplementary Table S3. Outcomes of subgroup analysis**

| **Cohorts** | **Test of association** | | | **Test of heterogeneity** | | | |
| --- | --- | --- | --- | --- | --- | --- | --- |
|  | **No. of studies** | **HR/OR/RR (95%CI)** | **P-value** | **Model** | **Chi-square** | **P-value** | **I²** |
| **Numbers of participants (OR values)** | | | | | | | |
| **Region/Country subgroup of risk** |  |  |  |  |  |  |  |
| Europe | 5 | 1.075 (0.953-1.212) | 0.242 | R | 9.23 | 0.056 | 56.70% |
| Iceland | 2 | 0.969 (0.845-1.110) | 0.647 | F | 0.55 | 0.460 | 0.00% |
| USA | 4 | 1.387 (0.897-2.145) | 0.141 | R | 107.27 | <0.001 | 97.20% |
| Asian | 2 | 1.142 (0.331-3.942) | 0.834 | R | 4.88 | 0.027 | 79.50% |
| **Netherlands** | **4** | **0.736 (0.600-0.904)** | **0.003** | **F** | **3.27** | **0.351** | **8.30%** |
| **Caucasian race** | **9** | **1.214 (1.041-1.415)** | **0.014** | **R** | **118.00** | **<0.001** | **93.20%** |
| **Lesion subgroup of risk** |  |  |  |  |  |  |  |
| **Hand** | **5** | **1.054 (1.029-1.080)** | **<0.001** | **F** | **3.52** | **0.475** | **0.00%** |
| Knee (or big joint) | 3 | 1.576 (0.861-2.883) | 0.140 | R | 54.84 | <0.001 | 96.40% |
| Small joint (hand and spine) | 6 | 1.104 (0.776-1.572) | 0.582 | R | 269.05 | <0.001 | 98.10% |
| **Study type subgroup of risk** |  |  |  |  |  |  |  |
| Case-control study | 2 | 1.015 (0.410-2.510) | 0.975 | R | 2.74 | 0.098 | 63.50% |
| **Cohort study** | **9** | **1.269 (1.016-1.584)** | **0.036** | **R** | **375.24** | **<0.001** | **97.90%** |
| **Follow-up subgroup of risk** |  |  |  |  |  |  |  |
| 2-5 years | 5 | 1.029 (0.931-1.136) | 0.578 | R | 8.22 | 0.084 | 51.40% |
| 2-6 years | 6 | 1.035 (0.970-1.104) | 0.302 | R | 11.47 | 0.043 | 56.40% |
| >2-year | 8 | 1.142 (0.923-1.413) | 0.222 | R | 287.05 | <0.001 | 97.60% |
| >4-year | 7 | 1.134 (0.903-1.427) | 0.276 | R | 286.86 | <0.001 | 97.90% |
| >5-year | 5 | 1.165 (0.808-1.679) | 0.413 | R | 267.34 | <0.001 | 98.50% |
| **>7-year** | **2** | **1.906 (1.782-2.039)** | **<0.001** | **F** | **0.99** | **0.320** | **0.00%** |
| **NOS** |  |  |  |  |  |  |  |
| 8 | 7 | 1.136 (0.903-1.427) | 0.276 | R | 286.86 | <0.001 | 97.90% |
| >8 | 8 | 1.108 (0.885-1.388) | 0.371 | R | 288.51 | <0.001 | 97.60% |
| <8 | 3 | 1.682 (0.989-3.861) | 0.055 | R | 31.97 | <0.001 | 93.70% |
| **Sample size** |  |  |  |  |  |  |  |
| **>3000** | **6** | **1.429 (1.097-1.861)** | **0.008** | **R** | **374.00** | **<0.001** | **98.70%** |
| <3000 | 5 | 0.993 (0.878-1.124) | 0.917 | F | 3.19 | 0.527 | 0.00% |
| **Adjusted Estimations** | | | | | | | |
| **Country** |  |  |  |  |  |  |  |
| USA | 3 | 1.228 (0.983-1.533) | 0.071 | F | 0.53 | 0.766 | 0.00% |
| Caucasian race | 10 | 1.045 (1.000-1.092) | 0.051 | F | 6.47 | 0.692 | 0.00% |
| Sweden | 4 | 1.046 (0.989-1.105) | 0.114 | R | 1.05 | 0.673 | 0.00% |
| UK | 3 | 1.024 (0.947-1.106) | 0.556 | F | 2.11 | 0.348 | 5.20% |
| Europe | 7 | 1.038 (0.992-1.086) | 0.103 | F | 3.84 | 0.698 | 0.00% |
| **Lesion** |  |  |  |  |  |  |  |
| Small (hand, spine and nodal) | 3 | 1.137 (0.922-1.403) | 0.230 | F | 28.60 | <0.001 | 93.00% |
| **Big (hip and knee)** | **7** | **1.056 (1.000-1.114)** | **0.049** | **F** | **3.96** | **0.683** | **0.00%** |
| **Confounders** |  |  |  |  |  |  |  |
| **Comorbidity adjusted** | **9** | **1.119 (1.002-1.248)** | **0.045** | **F** | **39.83** | **<0.001** | **79.90%** |
| Comorbidity unadjusted | 2 | 1.034 (0.923-1.157) | 0.486 | F | 1.90 | 0.168 | 47.40% |
| Co-medication adjusted | 5 | 1.040 (0.965-1.121) | 0.305 | F | 4.72 | 0.317 | 15.20% |
| Co-medication unadjusted | 6 | 1.115 (0.983-1.264) | 0.091 | F | 37.13 | <0.001 | 86.50% |
| Cigarette and/or alcohol consumption adjusted | 8 | 1.081 (0.976-1.198) | 0.135 | R | 47.93 | <0.001 | 85.40% |
| Cigarette and/or alcohol consumption unadjusted | 3 | 1.228 (0.983-1.533) | 0.071 | F | 0.53 | 0.766 | 0.00% |
| Other factors adjusted | 9 | 1.042 (0.997-1.090) | 0.069 | F | 5.51 | 0.702 | 0.00% |
| **Other factors unadjusted** | **2** | **1.346 (1.265-1.432)** | **<0.001** | **F** | **0.17** | **0.682** | **0.00%** |
| **Study type subgroup of risk** |  |  |  |  |  |  |  |
| **Cohort study** | **10** | **1.109 (1.007-1.222)** | **0.035** | **R** | **47.41** | **<0.001** | **81.00%** |
| **Follow-up subgroup of risk** |  |  |  |  |  |  |  |
| 2-5 years | 2 | 1.214 (0.915-1.610) | 0.178 | F | 0.52 | 0.472 | 0.00% |
| 5-6 years | 2 | 1.034 (0.923-1.157) | 0.486 | F | 1.90 | 0.168 | 47.40% |
| **>4-year** | **9** | **1.109 (1.002-1.226)** | **0.045** | **R** | **47.41** | **<0.001** | **83.10%** |
| >5-year | 8 | 1.100 (0.992-1.219) | 0.706 | R | 46.70 | <0.001 | 85.00% |
| >7-year | 6 | 1.123 (0.990-1.275) | 0.071 | R | 37.22 | <0.001 | 86.60% |
| **NOS** |  |  |  |  |  |  |  |
| 7 | 4 | 1.033 (0.957-1.114) | 0.406 | F | 3.24 | 0.356 | 7.40% |
| 8 | 6 | 1.127 (0.990-1.283) | 0.070 | R | 37.63 | <0.001 | 86.70% |
| **Sample size** |  |  |  |  |  |  |  |
| >10000 | 8 | 1.081 (0.976-1.198) | 0.135 | R | 47.93 | <0.001 | 85.40% |
| <10000 | 3 | 1.228 (0.983-1.533) | 0.071 | F | 0.53 | 0.766 | 0.00% |
