# Supplementary material for: The association between statin use and osteoarthritis-related outcomes: An updated systematic review and meta-analysis
Source: Front Pharmacol. 2022 Nov 24;13:1003370. doi: 10.3389/fphar.2022.1003370 (PMC9729269; doi:10.3389/fphar.2022.1003370)
Supplement: Supplementary file 6 [file Table4.DOCX]

**Supplementary data. Amendments to information provided from PROSPERO**

Our primary PROSPERO protocol was registered in April 28, 2020, due to COVID-19 pandemic and our own reasons, the PROSPERO protocol of this study was online updated and edited lastly in November 20, 2021, but website version was not updated yet, this version of study protocol may need to be further assessed by their editor team.

However, until recently we are keeping improving and amending the methodologies as the research going on. The last amendments in the protocol information of this study and reasons were listed below.

The last version of protocol of this study is available in the website:

<https://www.crd.york.ac.uk/prospero/display_record.php?ID=CRD42020163983>

**1. * Review title**

**Statement of last version:** The association between statin use and osteoarthritis-related outcomes: an updated systematic review and meta-analysis with more than 6 million participants.

**Current amendment:** The association between statin use and osteoarthritis-related outcomes: an updated systematic review and meta-analysis.

**Reason:** Revised statement may better and more concisely summarize this study.

**4. Anticipated completion date: Give the date by which the review is expected to be completed.**

**Statement of last version:** 20/03/2020

**Current amendment:** 20/05/2020

**Reason:** After screening the data and primary statistical analysis, we found it too great a workload to finish at anticipated date, then we had to apply for a delay in completing this study in our last request. The request for delaying this study was conducted by online updating in PROSPERO. Due to COVID-19 pandemic and our own reasons, this study was not completed on time. Until recently, we are keeping improving and amending the methodologies as the present study.

**5. * Stage of review at time of this submission.**

**Amendment:** We had already finished the works from preliminary searches to data analysis.

**Reason:** Before the day of the last edition online, we had finished the review.

**15. Review question.**

**Statement of last version:** To investigate whether statin use is associated with a higher or lower OA incidence and progression.

**Amendment:** To determine the associations between statin use and OA-related outcomes.

**Reason:** Revised statement may better and more concisely summarize this study as a multi-outcome research.

**16. Searches.**

**Statement of last version:** PubMed, Cochrane Library, Embase, Web of Science, Scopus databases will be comprehensively searched without any language restrictions, from database inception until January 1, 2020.

**Amendment:** PubMed, Cochrane Library, Embase, Web of Science, Scopus databases will be comprehensively searched without any language restrictions, from database inception until January 1, 2020, and updated lastly in June 1, 2022.

**Reason:** We updated the literature search as mentioned above and in the Method section.

**17. URL to search strategy**

**Statement of last version:** ((Statin OR *statin OR Atorvastatin OR cerivastatin OR crilvastatin OR Lovastatin OR mevastatin OR pitavastatin OR Pravastatin OR Rosuvastatin OR Simvastatin) AND (Osteoarthrit* OR osteo-arthrit* OR osteoarthro* OR osteo-arthro* OR “degenerative arthritis” OR osteoarthritis))

**Amendment:** We uploaded the detail search strategy.

**Reason:** We uploaded the detail search strategy with the aim of searching more comprehensively.

**18. * Condition or domain being studied.**

**Statement of last version:** Osteoarthritis (OA) is the most common arthritis and a major cause of pain and disability. Controversial conclusions are obtained from multiple previous studies regarding the associations between statin use and risk of OA incidence or progression. Therefore, a systematic review and meta-analysis would be helpful for drawing a conclusion on the relationship between statin use and OA incidence or progression.

**Amendment:** Osteoarthritis (OA) is the most common arthritis and a major cause of pain and disability. Controversial conclusions are obtained from multiple previous studies regarding the associations between statin use and risk of OA incidence or progression. Therefore, an updated systematic review and meta-analysis would be helpful for drawing a conclusion on the relationship between statin use and risk of OA-related outcomes.

**Reason:** We revised this statement because it was more precise to use the word “updated”.

**19. *Participants/population.**

**Statement of last version:** Statin users and non-users, a certain percentage of subjects in both groups may be with osteoarthritis. In individual or population-based studies.

**Amendment: *Population/Patient:*** participants using statins, controls were in the same population without statin medication prior identification;

**Reason:** This statement was revised to better summary the required characteristics of statin users and controls, as a part of PICOS inclusion.

**20. * Intervention(s), exposure(s).**

**Statement of last version:** Statin use, regardless of dosage, use duration, or kind. And variables of statin dosage, use duration may be further studied.

**Amendment:** Statin use

**Reason:** This statement was revised to be more concise.

**22. * Types of study to be included.**

**Statement of last version:** Studies like randomized controlled trials, cohort studies, cross-sectional studies, and case-control studies

**Amendment:** Clinical randomized/case-control/cohort studies

**Reason:** This statement was revised to be more concise.

**24. * Main outcome(s).**

**Statement of last version:** OR/HR/RRs of OA incidence or progression of the statin users versus non-users of population studied. Effect of dosage, duration of statin use on the OR/HR/RRs of OA incidence or progression.

**Amendment:** Data concerning OA risk or other related outcomes.

**Reason:** This statement was revised to be more concise and accurate.

**26. * Data extraction (selection and coding).**

**Statement of last version:** Studies selected will be based on the following criteria: (1) observational studies are concerning statin use and OA risk and progression; (2) participants with OA must be diagnosed and estimated with valid criteria; (3) studies with available data, otherwise only systematic review will be performed. Exclusion criteria are (1) non-observational studies (reviews, letters and case reports), and (2) studies irrelative with our interests. Data extraction of included studies will be performed by two investigators in duplicate independently and double-checked by a third investigator. Discordances will be addressed by open discussion with a senior investigator and resolved by consensus. We will extract the information like demographic (sex, age, data resource, etc.) and clinical data (lesion location, follow-up duration, etc.) of participants, outcomes and clinical significance, etc.

**Amendment:** Study selection was based on the PICOS statement. Population/Patient: participants with a prescription or a medication of statins, controls were in the same population without statin medication prior identification; Intervention: statin use; Comparison: OA risk or other OA-related outcomes; Outcome: data concerning OA risk or other related outcomes. Study design: clinical randomized/observational/case-control/cohort studies. From each included study, we extracted information such as demographic data, conclusions and clinical significances.

**Reason:** This statement was revised to be more concise and accurate.

**27.** *** Risk of bias (quality) assessment.**

**Statement of last version:** Methodological quality assessment of the included studies will be conducted independently by two investigators using predetermined criteria. Discordant judgments will be addressed by consensus discussion with a third investigator. For the included cross-sectional, case-control and cohort studies will be assessed using the Newcastle-Ottawa Scale (NOS) developed for these studies. RCTs will be assessed using the Cochrane risk-of-bias criteria.

**Amendment:** Risk of bias of included studies were assessed with the Newcastle-Ottawa Scale (NOS). **Reason:** This statement was revised to be more concise and accurate, due to no prospective RCT was included.

**28. * Strategy for data synthesis.**

**Statement of last version:** For the data eligible, meta-analysis will be performed using Stata 11.0 software. Detailed patient data, and OR/HR/RR of statin users vs. non-users will be used. Pooled data will be considered low heterogeneity if P is greater than 0.1 and I² is less than 50%. In these cases, a fixed effects model will be used; otherwise, a random effects model will be used. Statistical analyses will be 2-sided and a P-value less than 0.05 will be considered significant. The Z-score will be evaluated by the P-value of the 2-sided U-test for effect estimation. For the data ineligible for meta-analysis, we will list and analyze all the involved data without statistical synthesis.

**Amendment:** For the data eligible, meta-analysis will be performed using R 3.6.3 software. Detailed patient data, and OR/HR/RR of statin users vs. non-users will be used. Pooled data will be considered low heterogeneity if P is greater than 0.1 and I² is less than 50%. In these cases, a fixed effects model will be used; otherwise, a random effects model will be used. Statistical analyses will be 2-sided and a P-value less than 0.05 will be considered significant. The Z-score will be evaluated by the P-value of the 2-sided U-test for effect estimation. For the data ineligible for meta-analysis, we will list and analyze all the involved data without statistical synthesis.

**Reason:** This statement was revised due to we use R 3.6.3 software instead of Stata.

**33-34. Other registration details.**

**Statement of last version: -**

**Amendment:** INPLASY, Registration number: INPLASY202040160; URL: Zhang et al. Associations between statin use and osteoarthritis risk and progression: a systematic review and meta-analysis. Inplasy protocol 202040160. doi: 10.37766/inplasy2020.4.0160

**Reason:** We also registered the protocol of this study with INPLASY, the .pdf version of protocol was uploaded to PROSPERO.

**36. Keywords.**

**Statement of last version:** Statin, osteoarthritis, risk, progression, incidence

**Amendment:** Statin, Osteoarthritis, Meta-analysis, Risk, Antihypertensive drugs

**Reason:** We changed the keywords for concise statement of multi-outcome, antihypertensive drugs were detected being studied at the same time and were also analyzed.
