# Supplementary material for: The association between statin use and osteoarthritis-related outcomes: An updated systematic review and meta-analysis
Source: Front Pharmacol. 2022 Nov 24;13:1003370. doi: 10.3389/fphar.2022.1003370 (PMC9729269; doi:10.3389/fphar.2022.1003370)
Supplement: Supplementary file 9 [file Table2.doc]

**Supplementary Table S2. Outcomes of methodologic quality assessments**

**A. Outcomes of reference assessment (Newcastle-Ottawa Scale for Case-control Studies)**

| **First author** | **Selection** | | | | **Comparability** | | **Exposure** | | | **Total scores** |
| --- | --- | --- | --- | --- | --- | --- | --- | --- | --- | --- |
|  | **Is the case definition adequate? *** | **Representativeness of the cases†** | **Selection of Controls** | **Definition of Controls‡** | **Study controls for select the most important factor§** | **Study controls for any additional factor**** | **Ascertainment of exposure***** | **Same method of ascertainment for cases and controls** | **Non-Response rate****** |  |
| Frey | ★ | ★ | ★ | ★ | ★ | ★ | ★ | ★ | ★ | 9 |
| Valdes | ★ | - | - | ★ | ★ | ★ | ★ | ★ | ★ | 7 |
| Cemeroglu | ★ | ★ | ★ | ★ | ★ | - | ★ | ★ | ★ | 8 |

* A study with clear diagnosis from radiographic evidence or definite ICD code was assigned one star.

†A study with participants enrolled from large research projects and without biased selection was assigned one star.

‡A study with no significant difference in participants with OA between both groups in baseline was assigned one star.

§A study with no significant difference in factors like demographic factors, co-morbidity and medication, or study with confounders adjusted like the factors mentioned, or with PSM was assigned one star.

** A study with no significant difference in other factors like cigarette or alcohol consumption, or study with other confounders adjusted, or with PSM was assigned one star.

*** A study with a clear mean follow-up for 3 year or more was assigned one star.

****A study with a follow-up rate > 75% and almost equal non-response rate between groups was assigned one star.

**B. Outcomes of reference assessment (Newcastle-Ottawa Scale for Coho**rt Studies)

| **First author** | **Selection** | | | | **Comparability** | | **Exposure** | | | **Total scores** |
| --- | --- | --- | --- | --- | --- | --- | --- | --- | --- | --- |
|  | **Representativeness of the exposed cohort*** | **Selection of the non exposed cohort*** | **Ascertainment of exposure** | **Demonstration that outcome of interest was not present at start of study**† | **Study controls for the most important factor**‡ | **Study controls for any additional factor**§ | **Assessment of outcome** | **Was follow-up long enough for outcomes to occur**** | **Adequacy of follow up of cohorts***** |  |
| Eymard**** | ★ | ★ | ★ | ★ | ★ | ★ | ★ | ★ | ★ | 9 |
| Beattie | ★ | ★ | ★ | - | ★ | - | ★ | ★ | ★ | 7 |
| Jonsson | ★ | ★ | ★ | - | ★ | ★ | ★ | ★ | ★ | 8 |
| Chodick | ★ | ★ | ★ | - | ★ | ★ | ★ | ★ | ★ | 8 |
| Riddle | ★ | ★ | ★ | - | ★ | ★ | ★ | ★ | ★ | 8 |
| Kadam | ★ | ★ | ★ | - | ★ | - | ★ | ★ | ★ | 7 |
| Cheng | ★ | ★ | ★ | - | ★ | ★ | ★ | ★ | ★ | 8 |
| Garcia-Gil | ★ | ★ | ★ | - | ★ | ★ | ★ | ★ | ★ | 8 |
| Veronese | ★ | ★ | ★ | - | ★ | ★ | ★ | ★ | ★ | 8 |
| Burkard | ★ | ★ | ★ | - | ★ | ★ | ★ | ★ | - | 7 |
| Roy | ★ | ★ | ★ | - | - | - | ★ | - | ★ | 5 |
| Clockaerts | ★ | ★ | ★ | - | ★ | ★ | ★ | ★ | ★ | 8 |
| Michaelsson | ★ | ★ | ★ | - | ★ | ★ | ★ | ★ | ★ | 8 |
| Haj-Mirzaian | ★ | ★ | ★ | - | ★ | ★ | ★ | ★ | ★ | 8 |
| Cook | ★ | ★ | ★ | - | ★ | - | ★ | ★ | ★ | 7 |
| Sarmanova | ★ | ★ | ★ | - | ★ | ★ | ★ | ★ | ★ | 8 |
| Chaganti | ★ | ★ | ★ | - | ★ | - | ★ | - | ★ | 6 |
| Perry | ★ | ★ | ★ |  | ★ | ★ | ★ | ★ | ★ | 8 |
| Simic | ★ | ★ | ★ |  | ★ |  | ★ | ★ | ★ | 7 |
| Mansi | ★ | ★ | ★ | - | ★ | ★ | ★ | ★ | ★ | 8 |

*A Study with definite criteria of statin exposure and unexposure evidence was assigned one star.

†A Study with no significant difference in participants with OA between both groups in baseline was assigned one star.

‡ A Study with no significant difference in factors like demographic factors, co-morbidity and medication, or study with confounders adjusted like the factors mentioned, or with PSM was assigned one star.

§A Study with no significant difference in other factors like cigarette or alcohol consumption, or study with other confounders adjusted, or with PSM was assigned one star.

** A study with a clear mean follow-up for 3 year or more was assigned one star.

***A study with a follow-up rate > 75% and almost equal non-response rate between groups was assigned one star.

******** The study of Eymard was only used in progression cohort and the endpoint event was OA progression thus the item 4 was assigned a star.
